# Development of genetic tools for heterologous protein expression in a pentose‐utilizing environmental isolate of *Pseudomonas putida*

**DOI:** 10.1111/1751-7915.14205

**Published:** 2023-01-24

**Authors:** Rahul Gauttam, Thomas Eng, Zhiying Zhao, Qurrat ul ain Rana, Blake A. Simmons, Yasuo Yoshikuni, Aindrila Mukhopadhyay, Steven W. Singer

**Affiliations:** ^1^ The Joint BioEnergy Institute Emeryville California USA; ^2^ Biological Systems and Engineering Division Lawrence Berkeley National Laboratory Berkeley California USA; ^3^ Joint Genome Institute Berkeley California USA; ^4^ Environmental Genomics and Systems Biology Division Lawrence Berkeley National Laboratory Berkeley California USA

## Abstract

*Pseudomonas putida* has emerged as a promising host for the conversion of biomass‐derived sugars and aromatic intermediates into commercially relevant biofuels and bioproducts. Most of the strain development studies previously published have focused on *P. putida* KT2440, which has been engineered to produce a variety of non‐native bioproducts. However, *P. putida* is not capable of metabolizing pentose sugars, which can constitute up to 25% of biomass hydrolysates. Related *P. putida* isolates that metabolize a larger fraction of biomass‐derived carbon may be attractive as complementary hosts to *P. putida* KT2440. Here we describe genetic tool development for *P. putida* M2, a soil isolate that can metabolize pentose sugars. The functionality of five inducible promoter systems and 12 ribosome binding sites was assessed to regulate gene expression. The utility of these expression systems was confirmed by the production of indigoidine from C6 and C5 sugars. Chromosomal integration and expression of non‐native genes was achieved by using chassis‐independent recombinase‐assisted genome engineering (CRAGE) for single‐step gene integration of biosynthetic pathways directly into the genome of *P. putida* M2. These genetic tools provide a foundation to develop hosts complementary to *P. putida* KT2440 and expand the ability of this versatile microbial group to convert biomass to bioproducts.

## Graphical abstract

*Pseudomonas putida* has emerged as a promising host for the conversion of biomass‐derived sugars and aromatic intermediates into commercially relevant biofuels and bioproducts. Here we describe genetic tool development for *P. putida* M2, a soil isolate that can metabolize pentose sugars. These genetic tools provide a foundation to develop hosts complementary to *P. putida* KT2440 and expand the ability of this versatile microbial group to convert biomass to bioproducts.

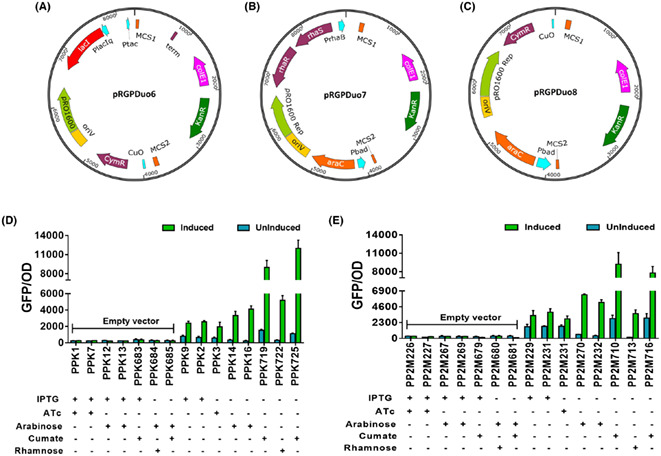

## INTRODUCTION

Lignocellulosic biomass is a globally available and sustainable carbon source for the production of biofuels and bio‐derived compounds (Bhatia et al., 2017; Cao et al., 2018). Lignocellulosic biomass is primarily composed of lignin (15%–20%), hemicellulose (25%–30%) and cellulose (30%–50%) (Menon & Rao, 2012; Zadeh et al., 2020). A critical step in the bioconversion of lignocellulosic biomass into bioavailable intermediates is chemical pre‐treatment of lignocellulose to increase enzymatic accessibility of the cellulose and hemicellulose biopolymers present in the plant cell wall (Bhatia et al., 2020; Brandt et al., 2013). However, the efficient bioconversion of cellulose and hemicellulose is still challenging and is not yet cost effective at the scales desired (Agbor et al., 2011). In this scenario, the use of microorganisms for lignin valorization is gaining significant attention. Most studies on lignin breakdown have primarily focused on fungi that utilize extracellular ligninolytic metalloenzymes to oxidize aromatic rings present in lignin (Bansal et al., 2012; Harindintwali et al., 2020). However, fungi are difficult to manipulate for protein expression using recombinant DNA technology (Bugg et al., 2021; Wilson, 2011). On the other hand, bacterial systems are easy to manipulate genetically to allow combinatorial strain improvement through pathway engineering (Bugg et al., 2021; Mueller et al., 2018; Pham et al., 2022). Most of the microorganisms possess the native pathways for the consumption of glucose, the most abundant sugar in lignocellulosic biomass (Kim & Woo, 2018). However, many other polysaccharides and their related monomeric sugars are also present in lignocellulosic biomass, and fewer microorganisms can metabolize these sugars in addition to glucose. For example, xylose is the second most abundant sugar in nature, and not many microorganisms possess the innate ability to metabolize xylose, thereby limiting the application of xylose (Domingues et al., 2021; Kim & Woo, 2018). Therefore, to improve the overall carbon conversion efficiency of fermenting microorganisms, engineering of these microbes to metabolize different sugars (such as xylose, arabinose, galactose) has become a crucial approach to generate value‐added compounds from lignocellulosic feedstocks (An et al., 2021; Elmore et al., 2020).

*Pseudomonas putida* KT2440 (hereafter, strain KT2440) is a promising bacterial chassis for the production of biofuels and bioproducts. KT2440 is best known for its versatile metabolism and tolerance to stresses (physiochemical and oxidative) and relatively toxic chemicals (e.g. ethanol, furfural, benzoate, acetic acid) that allow it to adapt in harsh environments (Fernández et al., 2009; Jayakody et al., 2018; Kim & Park, 2014). KT2440 exhibits many catabolic pathways that allow it to metabolize a wide range of molecules, including biomass‐derived aromatics, *p*‐coumarate and ferulate, that are present at high levels in the cell walls of grasses (Beckham et al., 2016; Linger et al., 2014). Since the model microbial hosts such as *Escherichia coli* cannot metabolize aromatic molecules, KT2240 has received significant attention as a potential bioconversion host that can convert glucose and aromatics. However, KT2440 cannot metabolize certain sugars (e.g. xylose, arabinose, galactose) that constitute most of the sugars in hemicellulose, therefore, limiting the utility of KT2440 as a bioproduction platform (Dvořák & de Lorenzo, 2018; Nogales et al., 2017; Puchałka et al., 2008; Rojo, 2010). KT2440 has been successfully engineered to utilize non‐native substrates such as xylose, arabinose, galactose, levoglucosan and cellobiose (Dvořák & de Lorenzo, 2018; Elmore et al., 2020; Linger et al., 2016; Peabody et al., 2019). However, the engineered strains often displayed slow growth phenotypes or had extended lag times (Elmore et al., 2020; Le Meur et al., 2012). An alternative approach to overcome these limitations shown by KT2440 is to explore different *Pseudomonas* strains with distinct characteristics. In the past, many strains related to KT2440 have been explored to produce chemicals based on idiosyncratic traits, such as: *P. putida* S16 (effective in nicotine degradation), *P. taiwanensis* VLB120 (native capability to utilize xylose but cannot utilize *p*‐coumarate), *P. putida* UV4 (converts toxic toluene into cis‐glycol), *P. putida* Fu1 (oxidize furfuryl alcohol to furfural) and *P. putida* S12 (converts HMF to HMF acid) (Hack et al., 1994; Hsu et al., 2020; Hu et al., 2019; Koenig & Andreesen, 1989; Köhler et al., 2015). Despite showing great potential, several limitations remain, and these promising strains are rarely used in biotechnological applications as compared to KT2440.

Here, we describe the development of genetic tools for *Pseudomonas putida* M2, an environmental isolate that can grow on glucose, xylose and plant‐derived aromatics (Park, Gauttam, et al., 2022). These tools include plasmid and chromosome‐based methods for heterologous protein expression that demonstrate production of indigoidine and flaviolin.

## EXPERIMENTAL PROCEDURES

### Bacterial strains, plasmids and culture conditions

Bacterial strains and plasmids used in this study are listed in Table 1 and Table S1, respectively. *Escherichia coli* DH5α (Hanahan, 1983) was used as the host strain for routine DNA manipulations such as subcloning and plasmid isolation. *E. coli* BW29427 (also known as WM3064) was used as a host for intergeneric conjugation to transfer DNA from *E. coli* to environmental isolates for CRAGE studies. Expression experiments were performed in KT2440 and environmental isolates M2. For general purposes, *E. coli* and *Pseudomonas* strains were cultured in Luria‐Bertani (LB) medium (tryptone 10 g/L, yeast extract 5 g/L, NaCl 2.5 g/L) at 37°C and 30°C, respectively. *Pseudomonas* and its derivatives were cultured in M9 minimal medium for expression and production experiments. M9 medium liquid was prepared using commercially available Difco M9 minimal salts 5X (Becton Dickinson, Franklin Lakes, NJ, USA) supplemented with 2 ml/L MgSO4.7H_2_O (1 M), 100 μmol/L calcium chloride, 1 ml/L trace elements (purchased from Teknova) and appropriate carbon source (C‐source; 0.5% w/v), namely glucose, xylose and arabinose. For the selection of plasmid‐containing transformants, the appropriate antibiotic was supplemented to the medium, namely, kanamycin (50 μg/ml), gentamicin (30 μg/ml) and apramycin (50 μg/ml). Inducers were used to induce gene expression or subsequent protein production at time *t* = 0 h. Unless otherwise stated, the following concentrations were used for inducers: for IPTG (1 mM), for L‐rhamnose (1 mM), 4‐isopropylbenzoic acid or cumate (1 mM), arabinose (0.2% w/v) and ATc (1000 ng/ml). All strains were grown on LB agar plates using cryo‐stocks for production experiments in *Pseudomonas*. A single colony was picked to inoculate 5 ml of LB medium (first preculture) and grown aerobically at 30°C with 200 rpm, until saturation. The inoculum from the first preculture was used for the second preculture, which contained M9 media (respective C‐source, no inducers) and was subsequently used to prepare the third preculture in the same C‐source containing media. The third preculture was used as an inoculum to inoculate the main culture (M9 medium) for production experiments and growth kinetics (and fluorescence experiments). All recombinant strains and plasmids used in this study have been deposited in the public instance of the JBEI Registry (http://public‐registry.jbei.org/) and are available on request.

**TABLE 1:** Strains used in this study.

Strain^a^	Relevant characteristics	Source / reference
*Strains*		
*E. coli* DH5α	F^−^ *φ*80*lacZ*∆M15 ∆(*lacZYA‐argF*) U169 *endA1 recA1 hsdR17* (r_k_^−^, m_k_^+^) *supE44 thi* ^*−1*^ *gyrA996 relA1 phoA*	Hanahan (1983)
*E. coli* BW29427	A conjugal donor strain to transfer plasmids, auxotrophic to diaminopimelic acid	Wang et al. (2019)
*P. putida* KT2440	Wild type	ATCC 12633
*P. putida* M2	Accession number JADOUD010000001	Park, Fong, et al. (2022)
*P. putida* PPK1 (PP1; JPUB_014489)	*P. putida* KT2440 carrying pRGPDuo2; Kan^R^	Gauttam et al. (2020)
*P. putida* PPK2 (PP2; JPUB_014491)	*P. putida* KT2440 carrying pRGPDuo2‐sfGFPtac RBS0; Kan^R^	Gauttam et al. (2020)
*P. putida* PPK3 (PP3; JPUB_014493)	*P. putida* KT2440 carrying pRGPDuo2‐sfGFPtet RBS0; Kan^R^	Gauttam et al. (2020)
*P. putida* PPK7 (PP7; JPUB_014775)	*P. putida* KT2440 carrying pRGPDuo1; Gent^R^	Gauttam et al. (2021)
*P. putida* PPK9 (PP9; JPUB_014779)	*P. putida* KT2440 carrying pRGPDuo1‐sfGFPtac RBS0; Gent^R^	Gauttam et al. (2021)
*P. putida* PPK12 (PP12; JPUB_014785)	*P. putida* KT2440 carrying pRGPDuo3; Gent^R^	Gauttam et al. (2021)
*P. putida* PPK13 (PP13; JPUB_014787)	*P. putida* KT2440 carrying pRGPDuo4; Kan^R^	Gauttam et al. (2021)
*P. putida* PPK14 (PP14; JPUB_014810)	*P. putida* KT2440 carrying pRGPDuo3‐sfGFPbad RBS0; Gent^R^	Gauttam et al. (2021)
*P. putida* PPK16 (PP16; JPUB_014791)	*P. putida* KT2440 carrying pRGPDuo4‐sfGFPbad RBS0; Kan^R^	Gauttam et al. (2021)
*P. putida* PP2M226 (JPUB_020213)	*P. putida* M2 carrying pRGPDuo1; Gent^R^	This study
*P. putida* PP2M227 (JPUB_020214)	*P. putida* M2 carrying pRGPDuo2; Kan^R^	This study
*P. putida* PP2M229 (JPUB_020215)	*P. putida* M2 carrying pRGPDuo1‐sfGFPtac RBS0; Gent^R^	This study
*P. putida* PP2M230	*P. putida* M2 carrying pRGPDuo2‐sfGFPtet RBS0; Kan^R^	This study
*P. putida* PP2M231 (JPUB_020216)	*P. putida* M2 carrying pRGPDuo2‐sfGFPtac RBS0; Kan^R^	This study
*P. putida* PP2M232 (JPUB_020217)	*P. putida* M2 carrying pRGPDuo4‐sfGFPbad RBS0; Kan^R^	This study
*P. putida* PP2M267 (JPUB_020218)	*P. putida* M2 carrying pRGPDuo3; Gent^R^	This study
*P. putida* PP2M269 (JPUB_020219)	*P. putida* M2 carrying pRGPDuo4; Kan^R^	This study
*P. putida* PP2M270 (JPUB_020220)	*P. putida* M2 carrying pRGPDuo3‐sfGFPbad RBS0; Gent^R^	This study
*P. putida* PP2M419 (JPUB_020221)	*P. putida* M2 carrying pRGPDuo1‐sfGFPtet + pRGPDuo4‐RFPbad; Gent^R^ + Kan^R^	This study
*P. putida* PP2M525 (JPUB_020430)	*P. putida* M2 carrying pRGPDuo2‐sfpbpsAtet; Kan^R^	This study
*P. putida* PP2M527 (JPUB_020432)	*P. putida* M2 carrying pRGPDuo4‐sfpbpsAbad; Kan^R^	This study
*P. putida* PPK529 (JPUB_020434)	*P. putida* KT2440 carrying pRGPDuo2‐sfpbpsAtet; Kan^R^	This study
*P. putida* PPK531 (JPUB_020222)	*P. putida* KT2440 carrying pRGPDuo4‐sfpbpsAbad; Kan^R^	This study
*P. putida* PP2M612 (JPUB_020222)	*P. putida* M2 carrying pRGPDuo2‐sfGFPtac RBS1; Kan^R^	This study
*P. putida* PP2M613 (JPUB_020224)	*P. putida* M2 carrying pRGPDuo2‐sfGFPtac RBS2; Kan^R^	This study
*P. putida* PP2M614 (JPUB_020226)	*P. putida* M2 carrying pRGPDuo2‐sfGFPtac RBS3; Kan^R^	This study
*P. putida* PP2M615 (JPUB_020228)	*P. putida* M2 carrying pRGPDuo2‐sfGFPtac RBS4; Kan^R^	This study
*P. putida* PP2M616 (JPUB_020230)	*P. putida* M2 carrying pRGPDuo2‐sfGFPtac RBS5; Kan^R^	This study
*P. putida* PP2M617 (JPUB_020232)	*P. putida* M2 carrying pRGPDuo2‐sfGFPtac RBS6; Kan^R^	This study
*P. putida* PP2M618 (JPUB_020234)	*P. putida* M2 carrying pRGPDuo2‐sfGFPtac RBS7; Kan^R^	This study
*P. putida* PP2M619 (JPUB_020236)	*P. putida* M2 carrying pRGPDuo2‐sfGFPtac RBS8; Kan^R^	This study
*P. putida* PP2M620 (JPUB_020238)	*P. putida* M2 carrying pRGPDuo2‐sfGFPtac RBS9; Kan^R^	This study
*P. putida* PP2M621 (JPUB_020240)	*P. putida* M2 carrying pRGPDuo2‐sfGFPtac RBS10; Kan^R^	This study
*P. putida* PP2M622 (JPUB_020242)	*P. putida* M2 carrying pRGPDuo2‐sfGFPtac RBS11; Kan^R^	This study
*P. putida* PP2M623 (JPUB_020244)	*P. putida* M2 carrying pRGPDuo4‐sfGFPbad RBS1; Kan^R^	This study
*P. putida* PP2M624 (JPUB_020246)	*P. putida* M2 carrying pRGPDuo4‐sfGFPbad RBS2; Kan^R^	This study
*P. putida* PP2M625 (JPUB_020248)	*P. putida* M2 carrying pRGPDuo4‐sfGFPbad RBS3; Kan^R^	This study
*P. putida* PP2M626 (JPUB_020250)	*P. putida* M2 carrying pRGPDuo4‐sfGFPbad RBS4; Kan^R^	This study
*P. putida* PP2M627 (JPUB_020252)	*P. putida* M2 carrying pRGPDuo4‐sfGFPbad RBS5; Kan^R^	This study
*P. putida* PP2M628 (JPUB_020254)	*P. putida* M2 carrying pRGPDuo4‐sfGFPbad RBS6; Kan^R^	This study
*P. putida* PP2M629 (JPUB_020256)	*P. putida* M2 carrying pRGPDuo4‐sfGFPbad RBS7; Kan^R^	This study
*P. putida* PP2M630 (JPUB_020258)	*P. putida* M2 carrying pRGPDuo4‐sfGFPbad RBS8; Kan^R^	This study
*P. putida* PP2M631 (JPUB_020260)	*P. putida* M2 carrying pRGPDuo4‐sfGFPbad RBS9; Kan^R^	This study
*P. putida* PP2M632 (JPUB_020262)	*P. putida* M2 carrying pRGPDuo4‐sfGFPbad RBS10; Kan^R^	This study
*P. putida* PP2M633 (JPUB_020264)	*P. putida* M2 carrying pRGPDuo4‐sfGFPbad RBS11; Kan^R^	This study
*P. putida* PPK634 (JPUB_020266)	*P. putida* KT2440 carrying pRGPDuo2‐sfGFPtac RBS1; Kan^R^	This study
*P. putida* PPK635 (JPUB_020267)	*P. putida* KT2440 carrying pRGPDuo2‐sfGFPtac RBS2; Kan^R^	This study
*P. putida* PPK636 (JPUB_020268)	*P. putida* KT2440 carrying pRGPDuo2‐sfGFPtac RBS3; Kan^R^	This study
*P. putida* PPK637 (JPUB_020269)	*P. putida* KT2440 carrying pRGPDuo2‐sfGFPtac RBS4; Kan^R^	This study
*P. putida* PPK638 (JPUB_020270)	*P. putida* KT2440 carrying pRGPDuo2‐sfGFPtac RBS5; Kan^R^	This study
*P. putida* PPK639 (JPUB_020271)	*P. putida* KT2440 carrying pRGPDuo2‐sfGFPtac RBS6; Kan^R^	This study
*P. putida* PPK640 (JPUB_020272)	*P. putida* KT2440 carrying pRGPDuo2‐sfGFPtac RBS7; Kan^R^	This study
*P. putida* PPK641 (JPUB_020273)	*P. putida* KT2440 carrying pRGPDuo2‐sfGFPtac RBS8; Kan^R^	This study
*P. putida* PPK642 (JPUB_020274)	*P. putida* KT2440 carrying pRGPDuo2‐sfGFPtac RBS9; Kan^R^	This study
*P. putida* PPK643 (JPUB_020275)	*P. putida* KT2440 carrying pRGPDuo2‐sfGFPtac RBS10; Kan^R^	This study
*P. putida* PPK644 (JPUB_020276)	*P. putida* KT2440 carrying pRGPDuo2‐sfGFPtac RBS11; Kan^R^	This study
*P. putida* PPK645 (JPUB_020277)	*P. putida* KT2440 carrying pRGPDuo4‐sfGFPbad RBS1; Kan^R^	This study
*P. putida* PPK646 (JPUB_020278)	*P. putida* KT2440 carrying pRGPDuo4‐sfGFPbad RBS2; Kan^R^	This study
*P. putida* PPK647 (JPUB_020279)	*P. putida* KT2440 carrying pRGPDuo4‐sfGFPbad RBS3; Kan^R^	This study
*P. putida* PPK648 (JPUB_020280)	*P. putida* KT2440 carrying pRGPDuo4‐sfGFPbad RBS4; Kan^R^	This study
*P. putida* PPK649 (JPUB_020281)	*P. putida* KT2440 carrying pRGPDuo4‐sfGFPbad RBS5; Kan^R^	This study
*P. putida* PPK650 (JPUB_020282)	*P. putida* KT2440 carrying pRGPDuo4‐sfGFPbad RBS6; Kan^R^	This study
*P. putida* PPK651 (JPUB_020283)	*P. putida* KT2440 carrying pRGPDuo4‐sfGFPbad RBS7; Kan^R^	This study
*P. putida* PPK652 (JPUB_020284)	*P. putida* KT2440 carrying pRGPDuo4‐sfGFPbad RBS8; Kan^R^	This study
*P. putida* PPK653 (JPUB_020285)	*P. putida* KT2440 carrying pRGPDuo4‐sfGFPbad RBS9; Kan^R^	This study
*P. putida* PPK654 (JPUB_020286)	*P. putida* KT2440 carrying pRGPDuo4‐sfGFPbad RBS10; Kan^R^	This study
*P. putida* PPK655 (JPUB_020287)	*P. putida* KT2440 carrying pRGPDuo4‐sfGFPbad RBS11; Kan^R^	This study
*P. putida* PPK656 (JPUB_020288)	*P. putida* M2 carrying pRGPDuo2‐sfGFPtet RBS1; Kan^R^	This study
*P. putida* PPK657 (JPUB_020290)	*P. putida* M2 carrying pRGPDuo2‐sfGFPtet RBS2; Kan^R^	This study
*P. putida* PPK658 (JPUB_020291)	*P. putida* M2 carrying pRGPDuo2‐sfGFPtet RBS3; Kan^R^	This study
*P. putida* PPK659 (JPUB_020293)	*P. putida* M2 carrying pRGPDuo2‐sfGFPtet RBS4; Kan^R^	This study
*P. putida* PPK660 (JPUB_020295)	*P. putida* M2 carrying pRGPDuo2‐sfGFPtet RBS5; Kan^R^	This study
*P. putida* PPK661 (JPUB_020297)	*P. putida* M2 carrying pRGPDuo2‐sfGFPtet RBS6; Kan^R^	This study
*P. putida* PPK662 (JPUB_020299)	*P. putida* M2 carrying pRGPDuo2‐sfGFPtet RBS7; Kan^R^	This study
*P. putida* PPK663 (JPUB_020301)	*P. putida* M2 carrying pRGPDuo2‐sfGFPtet RBS8; Kan^R^	This study
*P. putida* PPK664 (JPUB_020303)	*P. putida* M2 carrying pRGPDuo2‐sfGFPtet RBS9; Kan^R^	This study
*P. putida* PPK665 (JPUB_020305)	*P. putida* M2 carrying pRGPDuo2‐sfGFPtet RBS10; Kan^R^	This study
*P. putida* PPK666 (JPUB_020307)	*P. putida* M2 carrying pRGPDuo2‐sfGFPtet RBS11; Kan^R^	This study
*P. putida* PPK667 (JPUB_020309)	*P. putida* KT2440 carrying pRGPDuo2‐sfGFPtet RBS1; Kan^R^	This study
*P. putida* PPK668 (JPUB_020442)	*P. putida* KT2440 carrying pRGPDuo2‐sfGFPtet RBS2; Kan^R^	This study
*P. putida* PPK669 (JPUB_020310)	*P. putida* KT2440 carrying pRGPDuo2‐sfGFPtet RBS3; Kan^R^	This study
*P. putida* PPK670 (JPUB_020311)	*P. putida* KT2440 carrying pRGPDuo2‐sfGFPtet RBS4; Kan^R^	This study
*P. putida* PPK671 (JPUB_020312)	*P. putida* KT2440 carrying pRGPDuo2‐sfGFPtet RBS5; Kan^R^	This study
*P. putida* PPK672 (JPUB_020313)	*P. putida* KT2440 carrying pRGPDuo2‐sfGFPtet RBS6; Kan^R^	This study
*P. putida* PPK673 (JPUB_020314)	*P. putida* KT2440 carrying pRGPDuo2‐sfGFPtet RBS7; Kan^R^	This study
*P. putida* PPK674 (JPUB_020315)	*P. putida* KT2440 carrying pRGPDuo2‐sfGFPtet RBS8; Kan^R^	This study
*P. putida* PPK675 (JPUB_020316)	*P. putida* KT2440 carrying pRGPDuo2‐sfGFPtet RBS9; Kan^R^	This study
*P. putida* PPK676 (JPUB_020317)	*P. putida* KT2440 carrying pRGPDuo2‐sfGFPtet RBS10; Kan^R^	This study
*P. putida* PPK677 (JPUB_020318)	*P. putida* KT2440 carrying pRGPDuo2‐sfGFPtet RBS11; Kan^R^	This study
*P. putida* PP2M679 (JPUB_020319)	*P. putida* M2 carrying pRGPDuo6; Kan^R^	This study
*P. putida* PP2M680 (JPUB_020321)	*P. putida* M2 carrying pRGPDuo7; Kan^R^	This study
*P. putida* PP2M681 (JPUB_020323)	*P. putida* M2 carrying pRGPDuo8; Kan^R^	This study
*P. putida* PPK683 (JPUB_020325)	*P. putida* KT2440 carrying pRGPDuo6; Kan^R^	This study
*P. putida* PPK684 (JPUB_020326)	*P. putida* KT2440 carrying pRGPDuo7; Kan^R^	This study
*P. putida* PPK685 (JPUB_020327)	*P. putida* KT2440 carrying pRGPDuo8; Kan^R^	This study
*P. putida* PPK706 (JPUB_020328)	*P. putida* KT2440 (clone 1) carrying pW17 for landing pad integration at Locus tag PP_3007 that encodes a hypothetical protein, Kan^R^	This study
*P. putida* PP2M710 (JPUB_020330)	*P. putida* M2 carrying pRGPDuo6‐sfGFPCymR RBS0; Kan^R^	This study
*P. putida* PP2M711 (JPUB_020332)	*P. putida* M2 carrying pRGPDuo6‐sfGFPCymR RBS8; Kan^R^	This study
*P. putida* PP2M712 (JPUB_020334)	*P. putida* M2 carrying pRGPDuo6‐sfGFPCymR RBS10; Kan^R^	This study
*P. putida* PP2M713 (JPUB_020336)	*P. putida* M2 carrying pRGPDuo7‐sfGFPrhaRS RBS0; Kan^R^	This study
*P. putida* PP2M714 (JPUB_020338)	*P. putida* M2 carrying pRGPDuo7‐sfGFPrhaRS RBS8; Kan^R^	This study
*P. putida* PP2M715 (JPUB_020340)	*P. putida* M2 carrying pRGPDuo7‐sfGFPrhaRS RBS10; Kan^R^	This study
*P. putida* PP2M716 (JPUB_020342)	*P. putida* M2 carrying pRGPDuo8‐sfGFPCymR RBS0; Kan^R^	This study
*P. putida* PP2M717 (JPUB_020344)	*P. putida* M2 carrying pRGPDuo8‐sfGFPCymR RBS8; Kan^R^	This study
*P. putida* PP2M718 (JPUB_020346)	*P. putida* M2 carrying pRGPDuo8‐sfGFPCymR RBS10; Kan^R^	This study
*P. putida* PPK719 (JPUB_020348)	*P. putida* KT2440 carrying pRGPDuo6‐sfGFPCymR RBS0; Kan^R^	This study
*P. putida* PPK720 (JPUB_020349)	*P. putida* KT2440 carrying pRGPDuo6‐sfGFPCymR RBS8; Kan^R^	This study
*P. putida* PPK721 (JPUB_020350)	*P. putida* KT2440 carrying pRGPDuo6‐sfGFPCymR RBS10; Kan^R^	This study
*P. putida* PPK722 (JPUB_020351)	*P. putida* KT2440 carrying pRGPDuo7‐sfGFPrhaRS RBS0; Kan^R^	This study
*P. putida* PPK723 (JPUB_020352)	*P. putida* KT2440 carrying pRGPDuo7‐sfGFPrhaRS RBS8; Kan^R^	This study
*P. putida* PPK724 (JPUB_020353)	*P. putida* KT2440 carrying pRGPDuo7‐sfGFPrhaRS RBS10; Kan^R^	This study
*P. putida* PPK725 (JPUB_020354)	*P. putida* KT2440 carrying pRGPDuo8‐sfGFPCymR RBS0; Kan^R^	This study
*P. putida* PPK726 (JPUB_020355)	*P. putida* KT2440 carrying pRGPDuo8‐sfGFPCymR RBS8; Kan^R^	This study
*P. putida* PPK727 (JPUB_020356)	*P. putida* KT2440 carrying pRGPDuo8‐sfGFPCymR RBS10; Kan^R^	This study
*P. putida* PP2M729 (JPUB_020358)	*P. putida* M2 carrying pW17 for landing pad integration (clones 1–4); Kan^R^	This study
*P. putida* PPK757 (JPUB_020443)	*P. putida* KT2440 with CRAGE mediated RppA‐NT (RppA‐NW from *S. coelicolor* but with a C‐terminal truncation of 25 amino acids) integration #3; Apr^R^	This study
*P. putida* PP2M758 (JPUB_020444)	*P. putida* M2 with CRAGE mediated RppA‐NT integration #2; Apr^R^	This study
*P. putida* PP2M759 (JPUB_020445)	*P. putida* M2 with CRAGE mediated RppA‐NT integration #4; Apr^R^	This study

^a^ Strain name in bracket corresponds to the part ID assigned to each strain for JBEI public registry.

### General molecular biology procedures

All plasmids used in this study were constructed using standard cloning methods such as restriction enzyme‐based cloning (Thermo Fisher Scientific, Waltham, MA, USA) or HiFi DNA assembly (New England Biolabs), following the instructions from the manufacturer. Standard molecular biology techniques were carried out for DNA manipulations such as DNA isolation, ligation, electrophoresis, cloning, competent cell preparation and transformation (Green & Sambrook, 2012). The oligonucleotides used in this study are listed in Table S2 and ordered from Integrated DNA Technologies (IDT, San Diego, CA, USA). PCR conditions were optimized for each primer pair, and gene fragments were amplified using Q5 High‐Fidelity DNA polymerase (New England Biolabs, Ipswich, MA, USA). Colony PCR was performed to confirm cloning success using Taq polymerase (New England Biolabs, Ipswich MA, USA). Subsequently, the selected clones were sequence‐verified using the Sanger Sequencing service (Genewiz, CA, USA). Commercial kits from Qiagen (Hilden, Germany) were used to isolate recombinant plasmids from *E. coli* and to separate PCR products (or concentrate DNA) from agarose gels (1% w/v). Primer designing (SnapGene, GSL Biotech; available at snapgene.com), vector map generation (SnapGene), graph preparation (GraphPad Prism) and gene analysis (NCBI Blast) were performed using basic bioinformatics tools.

### Electroporation

For most experiments, plasmids were transferred to the recipient *Pseudomonas* strain by electroporation, using a 2‐mm cuvette on a Gene Pulser XCell™ (BioRad Labs GmbH, Munich, Germany). The respective *Pseudomonas* strain was grown overnight on LB agar plates to prepare competent cells. Cells were resuspended in 300 mM sucrose solution and washed three times with sucrose solution before making 100 μl aliquots and storing them at −80°C. Before electroporation, the cells were thawed (on ice), and plasmid DNA was added (up to 1 μg), followed by electric shock using an electroporator with parameters set to voltage 2.5 kV capacitance of 25 μF, and resistance of 200 Ω. Immediately, cells were resuspended in 2 ml of LB media. Cells were allowed to recover for 2 h at 30°C with 200 rpm on a shaker incubator before plating LB plates with the respective antibiotic(s).

### Chromosomal integration of landing pad and reporter gene (rppA)

Conjugation was used to establish chassis‐independent recombinase‐assisted genome engineering (CRAGE) since electroporation proved to be unsuccessful. Plasmids for CRAGE‐mediated integration into the chromosome were constructed in donor *E. coli* BW29427 strain (hereafter BW29427) and conjugated into *Pseudomonas* strains. BW29427 is a diaminopimelic acid (DAP) auxotroph; therefore, its growth requires 0.3 mM DAP even when grown in LB (Wang et al., 2019; Zhao et al., 2022). For landing pad (LP) integration, BW29427 harbouring pW17 was the conjugal donor strain. The donor strain (BW29427 with 0.3 mM DAP) and recipient strain (*Pseudomonas*) were grown overnight in a shaker incubator at 37°C and 30°C, respectively. The next day, 1.5 ml cells from donor and recipient were washed three times with LB medium supplemented with 0.3 mM DAP to remove antibiotic residues. To increase the efficiency of *Pseudomonas* for conjugation, the cells were given heat shock for 15 min at 42°C. After that donor and recipient were mixed in a 4:1 ratio with a final volume of 100 μl. Cells were spinned down and resuspended 50 μl LB containing 0.3 mM DAP. This resuspended mix was then placed on LB agar plates (without antibiotic) containing 0.3 mM DAP. The plate was incubated for 1 to 2 days at 30°C. Following incubation, the spot was restreaked onto a *Pseudomonas* isolation agar (PIA) plate supplemented with kanamycin (without DAP). This step ensures the growth of only successful *Pseudomonas* transformants harbouring landing pads since *E. coli* cannot survive on PIA (and also without DAP). Landing pad integration was confirmed with colony PCR using two set of primers (Wang et al., 2019), LPdetection_fwd/rev (for LP detection) and pW17detection_fwd/rev (for pW17 detection).

The second round of conjugation was performed to deliver a pW34‐derived plasmid to assist in CRAGE mediated chromosomal integration of reporter gene (*rppA*). For this purpose, a pW34‐based construct was used in the BW29427 host background. Conjugation was performed on a similar outline to LP integration, and the bacterial mix was streaked on PIA plates supplemented with apramycin (without DAP) for selection. For final screening and to confirm removal of pW17 backbone, clones were simultaneously streaked on LB with kanamycin and LB with apramycin. The strains that grew on apramycin plates and not on kanamycin plates were the strains of interest with successful gene integration into the chromosome. Chromosomal integration of pW34‐derived vectors was confirmed using antibiotic selection and subsequent flaviolin quantification. The details regarding the construction of pW34‐derived vectors are described in Supporting Information.

### PacBio sequencing

The chromosomal integration of landing pad plasmid pW17 at a specific location in the genome was confirmed for using PacBio sequencing. The 10 ml cells were grown in LB media, and genomic DNA was isolated using Qiagen DNeasy Blood and Tissue Kit (Hilden, Germany) following the instructions from the manufacturer. Isolated genomic DNA (~5 μg) was sent to DOE Joint Genome Institute for PacBio Sequencing. Integration of pW17 was verified by the PacBio RSII system (Pacific Biosciences) with default settings and parameters (Hatmaker et al., 2020; Wang et al., 2019). PacBio sequencing confirmed the integration locus in KT2440 (PPK706, clones 1–4) but integration locus could not be confirmed for M2 (PP2M729, clones 1–4) due to multiple sequence alignments to pW17.

### Construction of rhamnose and cumate‐ inducible duet‐expression vectors pRGPDuo6, pRGPDuo7 and pRGPDuo8

**FIGURE 1:**
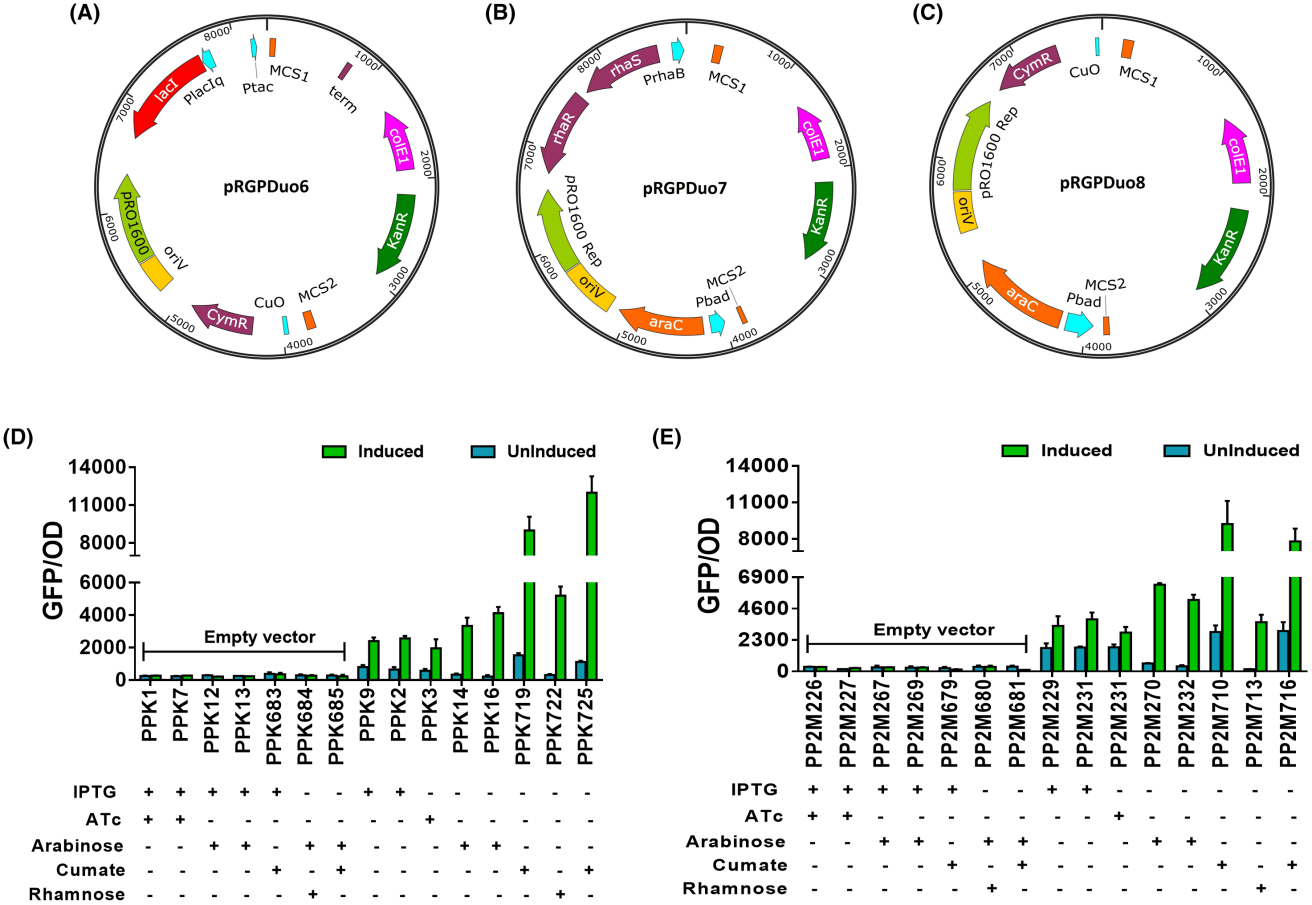
Plasmid maps of dual‐inducible duet‐vectors pRGPDuo6 (A), pRGPDuo7 (B) and pRGPDuo8 (C). The recombinant *P. putida* KT2440 (D) and M2 (E) strains were investigated for GFP fluorescence in a minimal medium with glucose (0.5% w/v) as C‐source. Functionality of five promoters, P_*tac*_ (1 mM IPTG), P_*tetR/tetA*_ (1 μg/ml ATc), P_*bad*_ (0.2% arabinose), rhaP_*bad*_ (1 mM rhamnose), P_*veg/Cuo*_ (1 mM cumate) were assessed in KT2440 and M2. The inducers were added at *t* = 0 h. the graphs consist of relative fluorescence units normalized to OD_600_ during the mid‐exponential phase for each recombinant strain. Levels of fluorescence for induced (green bar in graphs) and uninduced (no inducer was added, the blue bar in graphs) cultures are shown. The strains harbouring empty vectors were used as negative control and uninduced counterparts for determining background fluorescence. The (+) sign below each strain is indicative for inducer(s) added to induce reporter gene expression driven by corresponding promoter system and (−) sign is indicative of absence of inducer. The initial letters in recombinant strains name are representative of host background, PPK for ‘KT2440’ and PP2M for ‘M2’. For strain description and promoter‐RBS combination, refer to Table 1. Data represent mean values of triplicate assays from at least three individual cultivations, and error bars represent standard deviations.

For constructing expression plasmids pRGPDuo6 (Figure 1A), the previously described expression vector pRGPDuo2 was used as the parent vector (Gauttam et al., 2020). The DNA fragment containing the nucleotide sequence coding for cumate‐inducible repression machinery (CymR and P_*veg/Cuo*_ promoter) was amplified with incorporated restriction sites from pCT5bac2.0 (Seo & Schmidt‐Dannert, 2019) using 55/56cumateduo2 fwd/rev primers. Then, the EcoRI/BglII‐digested amplified fragment was ligated into MunI/BglII‐digested pRGPDuo2 to construct pRGPDuo6.

To construct the expression plasmids pRGPDuo7 (Figure 1B) and pRGPDuo8 (Figure 1C), expression plasmid pRGPDuo4 was used as the parent vector. Gibson assembly method (NEB) was employed to generate these constructs. It is noteworthy that the functionality of rhamnose‐inducible promoter, rhaP_*bad*_, requires the expression of two activators, RhaR and RhaS. To create pRGPDuo7, the DNA fragment containing the nucleotide sequence coding for rhamnose‐inducible repression machinery (*rhaR*, *rhaS* and rhaP_*bad*_) were amplified from pJeM1 (Jeske & Altenbuchner, 2010) using primers pair 57/58rhaRduo4 fwd/rev and 59/60rhaSduo4 fwd/rev. The amplified fragments were assembled and ligated into MunI/BglII‐digested pRGPDuo4 using HiFi DNA assembly master mix to create pRGPDuo7. Similarly, to create pRGPDuo8, the DNA fragment containing the nucleotide sequence coding for cumate‐inducible repression machinery (CymR and P_*veg/Cuo*_ promoter) was amplified from pCT5bac2.0 using primers 61/62cumateduo4 fwd/rev. The amplified fragment was ligated into BsiWI/PstI digested pRGPDuo4 using HiFi DNA assembly master mix to create pRGPDuo8. Likewise, their parent vectors, these vectors were designed to retain the necessary feature to be stably maintained in *P. putida* KT2440 when co‐transformed with pBBR1‐derived vectors. The details regarding the construction of pRGPDuo‐derived vectors are mentioned in Supporting Information.

### Measurement of sfGFP and RFP fluorescence

The cells adapted in M9 minimal media (refer to growth section above) were used to prepare 48‐well plates (Sarstedt, Germany). If required, each well contained 250 μl of cell culture with appropriate antibiotic and inducer combination. The GFP fluorescence was measured relative to the optical density (OD_600_) using a Synergy plate reader (Biotek Instruments, Inc, VT, USA). Similarly, RFP fluorescence was measured relative to the optical density (OD_600_) using a spectrofluorophotometer TECAN infinite M200 PRO reader (Mannedorf, Switzerland). The relative fluorescence was obtained by dividing the GFP fluorescence by the corresponding OD_600nm_. The cells were grown at 30°C with continuous shaking for the next 24–72 h (substrate dependent) on a spectrofluorophotometer, and fluorescence relative to OD_600nm_ was recorded at an interval of every 15 min. An excitation wavelength of 485 nm and an emission wavelength of 535 nm was used for sfGFP, and an excitation wavelength of 575 nm and an emission wavelength of 620 nm was used for RFP. The cultivation experiments were performed in triplicate.

### Indigoidine quantification

For indigoidine quantification, a previously established protocol was followed (Lim et al., 2021). The samples were taken directly from 5 ml culture tubes after ~16 h (at the late exponential phase). The absorbance (OD_612nm_) was measured using a plate reader from BD Biosciences (Molecular Devices, CA, USA).

### Flaviolin production and quantification

The tetrahydroxynaphthalene synthase (RppA) from *Streptomyces coelicolor* was utilized to develop malonyl‐CoA biosensor in *P. putida* (Yang et al., 2018). RppA catalyses the conversion of malonyl‐CoA to an intermediate product 1,3,6,8‐tetrahydroxynaphthalene that spontaneously oxidizes to red‐coloured pigment molecule flaviolin that can be quantified at 340 nm. CRAGE technology was applied to integrate *rppA* gene to produce flaviolin in environmental isolates. For this purpose, the nucleotide sequence coding for *S. coelicolor rppA* (3′ truncation of 75 base pairs) was amplified with incorporated restriction sites using 101/102CRAGErppANT fwd/rev primers. The NdeI/NheI‐digested amplified fragment (containing *rppA*) was ligated into NdeI/NheI‐digested CRAGE accessory vector pW34 to construct pW34‐*rppA*‐NT that was conjugated with strains harbouring landing pad to integrate *rppA* gene into the genome. For endpoint measurements, samples were taken directly from 5 ml grown culture in 30 ml tubes after ~16 h (at late exponential phase). The absorbance (OD_340nm_ and OD_600nm_) was measured using a plate reader from BD Biosciences (Molecular Devices, CA, USA). Flaviolin levels were quantified by normalizing the OD_340nm_ with the corresponding OD_600nm_.

## RESULTS

### Evaluation of inducible gene expression in P. putida M2

Inducible repressor‐promoter systems are useful tools for metabolic engineering to control heterologous gene expression, particularly when this expression may inhibit growth. Several promoters, including P_*tac*_ (IPTG‐inducible), P_*tetR/tetA*_ (ATc‐inducible), P_*bad*_ (arabinose‐inducible), rhaP_*bad*_ (rhamnose‐inducible), P_*veg/Cuo*_ (cumate‐inducible) have been characterized in strain KT2440 (Calero et al., 2016; Eaton, 1997; Gauttam et al., 2021). The behaviour of these promoters was tested in strain M2. The utility of P_*tac*_, P_*tetR/tetA*_, P_*bad*_, was previously demonstrated as part of a dual‐inducible duet‐expression system (pRGPDuo1–4) in KT2440, and all these systems were found to exhibit some level of constitutive expression (Gauttam et al., 2021). To find more tightly regulated gene expression plasmids, the pRGPDuo vector series was expanded to include L‐rhamnose‐inducible (pRGPDuo7) and cumate‐inducible (pRGPDuo6 and pRGPDuo8) promoter systems. The cumate‐inducible repression machinery (CymR and P_*veg/Cuo*_ promoter) was derived from pCT5bac2.0 (Seo & Schmidt‐Dannert, 2019) and rhamnose‐inducible repression machinery (*rhaR*, *rhaS* and rhaP_*bad*_) was derived from pJeM1 (Jeske & Altenbuchner, 2010). The functionality of these expression vectors was first confirmed in KT2440 (Figure 1D). The gene encoding for sfGFP was cloned into pRGPDuo1‐8 vectors, and resulting plasmids were transformed into KT2440 to generate empty vector harbouring control strains and sfGFP‐plasmid harbouring test strains. In KT2440, GFP expression increased in test strains by 3 to 40‐fold compared to their uninduced counterparts and by 9 to 40‐fold compared to control strains on induction (Figure 1D). An increase in fluorescence in all test strains established the functional validation of pRGPDuo1‐8 vector series. Subsequently, the same plasmids were transformed into M2 to generate empty vector harbouring control strains and sfGFP‐plasmid harbouring test strains. In M2, GFP expression increased in test strains by 2 to 22‐fold compared to their uninduced counterparts and 9 to 26‐fold compared to control strains on induction (Figure 1E). The pattern regarding promoter strength and leakiness was quite similar among KT2440 and M2. The strongest GFP expression was observed in both bacterial hosts from P_*veg/Cuo*_ followed by P_*bad*_ ≥ rhaP_*bad*_ > P_*tac*_ ≥ P_*tetR/tetA*_. The rhamnose‐inducible promoter was not leaky, and the fluorescence levels in the uninduced PP2M713 and PPK722 strains were comparable to the control strains. In contrast, other promoters showed leakiness to a different extent, with P_*veg/Cuo*_ being the leakiest one followed by P_*tac*_ ≥ P_*tetR/tetA*_ > P_*bad*_ (Figure 1D,E). Taken together, our data indicate that these five promoters could be used to modulate the expression of genes for new isolate M2.

### Characterization of RBS library

**FIGURE 2:**
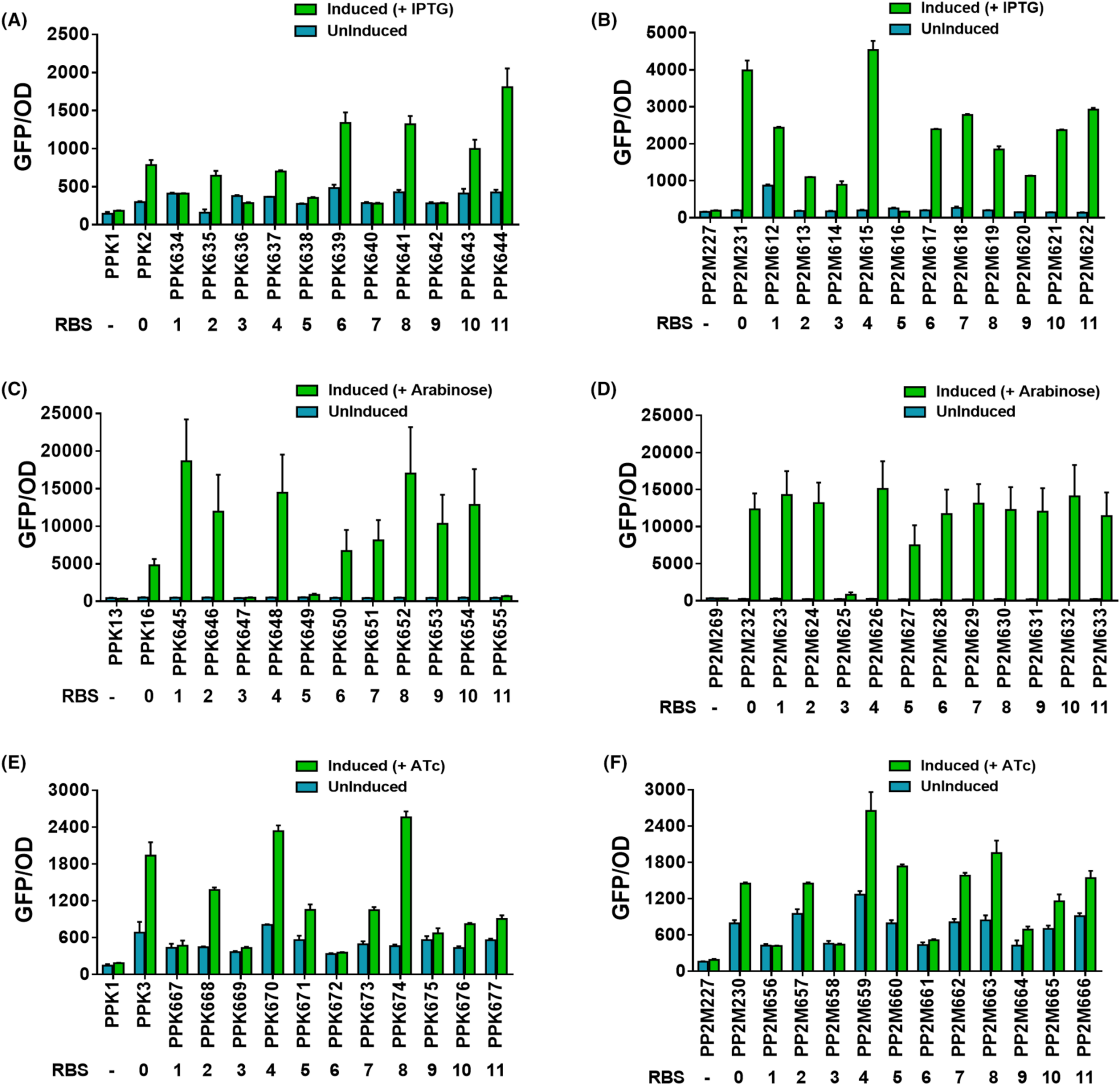
Comparative strengths of ribosome binding sites (RBSs). The selected RBS sequences (1 to 11) were taken from literature (Wang et al., 2018) and previously designed to modulate gene expression in *P. putida* based on Salis RBS calculator to cover a wide spectrum of expression levels. GFP activity assay was performed to compare the translational efficiency of twelve different RBSs (0 to 11) in *P. putida* KT2440 (A, C, E) and M2 (B, D, F) in M9 minimal media supplemented with glucose (0.5% w/v). All recombinant strains (harbouring RBS plasmids) were induced with respective promoter systems, namely, P_*tac*_ (a, B), P_*bad*_ (C, D) and P_*tetR/tetA*_ (E, F) at *t* = 0 h. Levels of fluorescence for induced (green bar in graphs) and uninduced (no inducer was added, the blue bar in graphs) cultures are shown. The strains harbouring empty vectors were used as negative control and uninduced counterparts for determining background fluorescence. The number below each strain represents the corresponding RBS sequence under the control of a specific promoter system (P_*tac*_, P_*bad*_ and P_*tetR/tetA*_). The (−) sign is indicative of empty vector, therefore, no RBS. For strain description and promoter‐RBS combination, refer to Table 1. Data represent mean values of triplicate assays from at least three individual cultivations, and error bars represent standard deviations.

**FIGURE 3:**
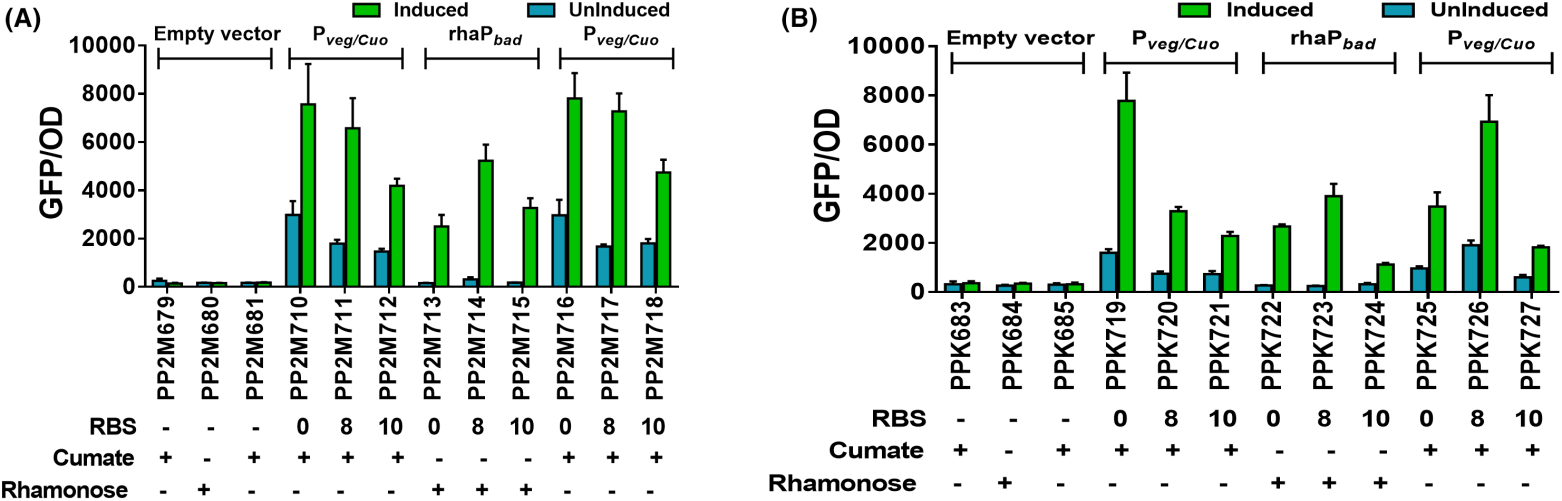
Comparative strengths of three shortlisted RBSs with promoters rhaP_*bad*_ (1 mM rhamnose) and P_*veg/Cuo*_ (1 mM cumate) in M2 (A) and KT2440 (B). GFP levels were measured by growing strains in a minimal medium with glucose as C‐source with respective antibiotics. The inducers were added at *t* = 0 h. the graphs consist of relative fluorescence units normalized to OD_600_ during the mid‐exponential phase for each recombinant strain. Levels of fluorescence for induced (green bar in graphs) and uninduced (no inducer was added, the blue bar in graphs) cultures are shown. The strains harbouring empty vectors were used as negative control and uninduced counterparts for determining background fluorescence. The number below each strain represents the corresponding RBS sequence (0, 8 and 10) under the control of a specific promoter system (P_*veg/Cuo*_ and rhaP_*bad*_). The (+) sign below each strain corresponds to inducer added to induce gene expression and (−) sign corresponds to either the empty vector (without any RBS) or absence of inducer. For strain description, a promoter‐RBS combination refers to Table 1. Data represent mean values of triplicate assays from at least three individual cultivations, and error bars represent standard deviations.

Besides promoters, protein expression can be modulated by many factors, including ribosome binding site (RBS), start codon sequence, −10/−35 sequences and spacing. The combination of an appropriate promoter and an RBS can significantly impact protein production. The above‐described constructs harboured a strong bacterial RBS (hereafter RBS0) that had the canonical sequence 5′‐AAAGAGGAGAA‐3′ and was spaced six nucleotides upstream of the start codon for each reporter gene (Lucidi et al., 2019). To further optimize protein expression in our pRGPDuo vector series, we selected 11 RBS sequences (Table S3) previously characterized in KT2440 (Wang et al., 2018) and compared their expression in M2 with three different promoter systems. These 11 RBS sequences were designed based on an in‐silico RBS calculator and were predicted to cover a wide spectrum of expression levels in *P. putida* ATCC 12633 (Wang et al., 2018). The sequence for selected RBSs was cloned upstream of sfGFP to generate many plasmids, which were subsequently transformed into KT2440 (PPK‐derivatives) and into M2 (PP2M‐derivatives) to develop recombinant strains: PPK634–644 (IPTG‐inducible), PP2M612–622 (IPTG‐inducible), PPK645–655 (arabinose‐inducible), PP2M623–633 (arabinose‐inducible), PPK667–677 (ATc‐inducible), PP2M656–666 (ATc‐inducible). To evaluate the effect of RBSs on protein expression, we used strains harbouring empty vectors (negative control) and strains carrying RBS0 (positive control) as reference for RBS strength. All GFP expressing RBS‐constructs were tested in KT2440 and M2. As expected, GFP expression was not observed in control strains harbouring empty vectors (Figure 2A–F). The 12 RBSs showed a considerable variation in expression strength for both KT2440 and M2 when induced with the respective inducer (IPTG, arabinose and ATc). Interestingly, in both hosts, the RBS‐specific expression pattern was consistent for a few RBSs in reference to a distinct inducible promoter system (Figure 2A–F). For example, RBS0, RBS4, RBS8, RBS10 and RBS11, consistently demonstrated strong expression, whereas RBS3 consistently demonstrated the weakest expression with all three inducible systems in KT2440 and M2 when grown in M9 medium supplemented with glucose as C‐source (Figure 2A–F). Similar results were obtained when M2‐derived strains were grown in M9 media supplemented with xylose or arabinose as C‐source (Figures S1 and S2). Again, RBS0, RBS4, RBS8, RBS10, RBS11 demonstrated more robust expression, whereas RBS3 demonstrated the weakest expression (Figures S1 and S2). We also investigated the RBSs strength with cumate‐ and rhamnose‐inducible promoters using pRGPDuo6‐8 vectors (Figure 3A,B). We tested the three RBSs, RBS0, RBS8 and RBS10, for this set of experiments that consistently worked best in both hosts with all tested inducible systems. The plasmids harbouring a particular RBS under the control of respective promoters were transformed in KT2440 (PPK‐derivatives) and M2 (PP2M‐derivatives) to generate recombinant strains: PPK719‐727 and PP2M710–718, respectively. The vectors harbouring empty vectors were used as control. Again, the RBSs showed variation in expression strength for both the hosts (Figure 3A,B). All three RBSs showed stronger expression in M2 with both inducible promoter systems, whereas in KT2440, RBS0 provided strongest expression in pRGPDuo6, RBS8 in both pRGPDuo7 as well as in pRGPDuo8 (Figure 3A,B).

### Compatibility of pBBR1‐ and pRO1600‐based pRGPDuo vectors in P. putida M2

**FIGURE 4:**
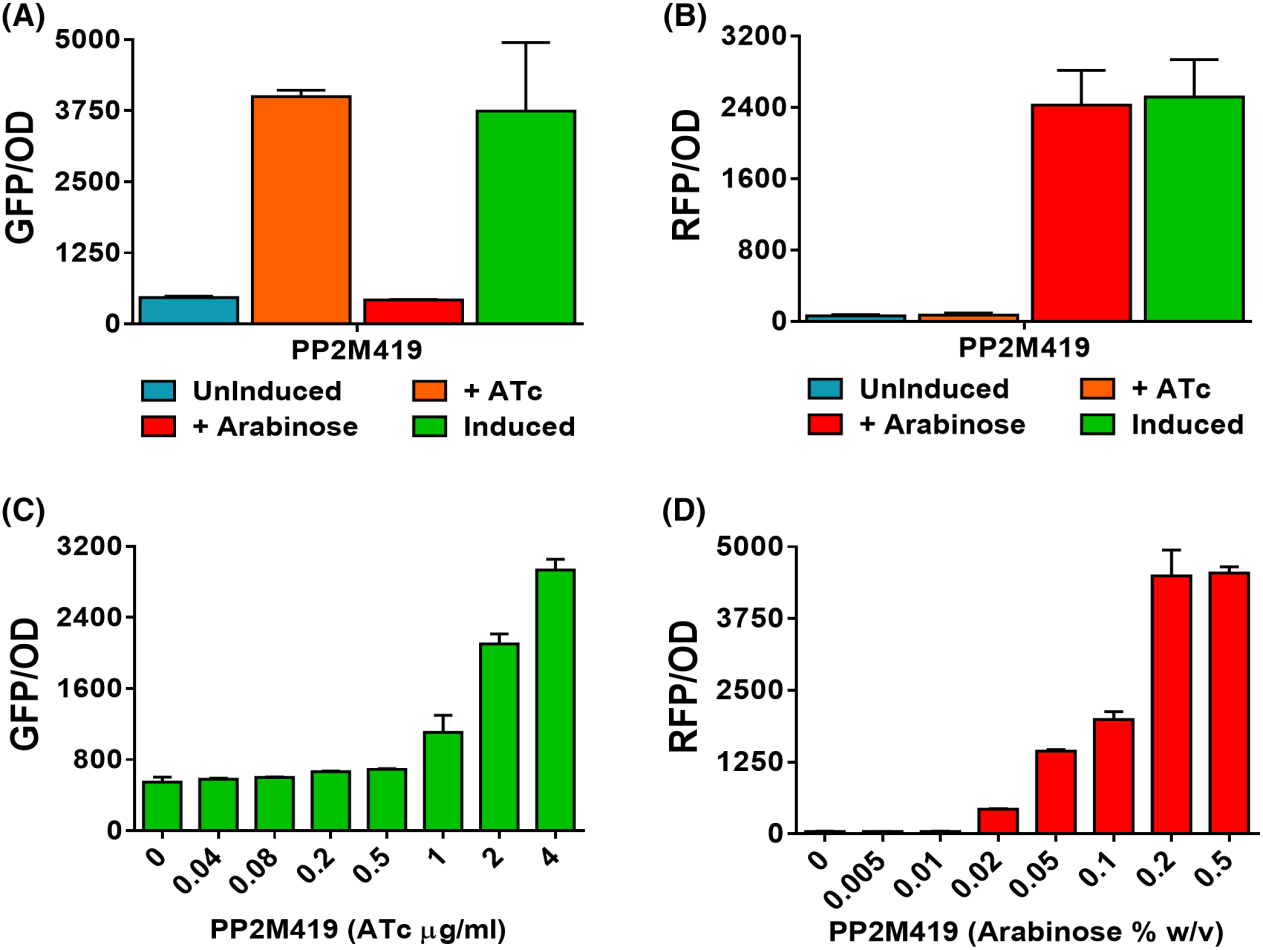
Checking the compatibility of pBBR1‐derived plasmids with pRO1600‐derived plasmids in M2 (A, B). GFP and RFP levels were investigated for recombinant strain, PP2M419 (M2 harbouring plasmids pRGPDuo1‐sfGFP_tet_ + pRGPDuo4‐RFP_bad_). The GFP (a) and RFP (B) expression levels measured in the presence of a respective cognate inducer(s) are shown. The inducers were added at *t* = 0 h. The annotation indicates the presence or absence of inducers: Uninduced (no inducer added); induced (presence of both inducers: Combination of 1 μg/ml ATc and 0.2% w/v arabinose), +ATc (only ATc was added), +arabinose (only arabinose was added). GFP (C) and RFP (D) levels were also measured in strain PP2M419 with serial concentrations of specific inducer, namely, ATc (ranging from 0 to 4 μg/ml) and arabinose (ranging from 0 to 0.5% w/v). The graphs consist of relative fluorescence units normalized to OD_600_ during the mid‐exponential phase for each construct. Data represent mean values of triplicate assays from at least two individual cultivations, and error bars represent standard deviations.

In our previous study, we have demonstrated the compatibility of pBBR1‐ and pRO1600‐derived plasmids in KT2440 for co‐expression studies (Gauttam et al., 2021). Like KT2440, the new isolate M2 is sensitive to kanamycin, gentamicin and apramycin (Figure S3). Following the experimental setup from our previous study (Gauttam et al., 2021) we tested the compatibility of pBBR1‐ and pRO1600‐derived plasmids in M2. For this purpose, a strain PP2M419 was generated by co‐transforming two plasmids possessing distinct selection markers and possessing ori(s) from different incompatibility groups. The cultivation of recombinant strain PP2M419 in presence of both antibiotics ensures the stable maintenance of both plasmids inside the cell. The reporter protein expression was evaluated under three conditions (a) no inducer was added (Uninduced), (b) both inducers were added (Induced), and (c) only one inducer was added (either ATc or arabinose). In PP2M419, GFP and RFP expression were minimal when none of the inducers was added (uninduced), and the levels of both reporter proteins increased in the presence of inducers ATc and arabinose (Figure 4A,B). Also, in the presence of a single inducer, GFP and RFP were produced only in the presence of respective cognate inducer, that is RFP in the presence of arabinose, and GFP in the presence of ATc (Figure 4A,B). In contrast, the fluorescence levels in the presence of non‐cognate inducers (ATc for RFP and arabinose for GFP) in PP2M419 were in the same range as that of uninduced cultures. The expression of both reporter proteins in the same strain ensures the functionality of compatible plasmids.

The induction range for inducible promoter systems ATc (ranging from 0 to 4 μg/ml), and arabinose (ranging from 0 to 0.5% w/v) was tested to check the tunability of these expression plasmids in strain M2. GFP and RFP expression was quantified in PP2M419 with varying ATc (fixed 0.2% arabinose; Figure 4C) and varying arabinose (fixed 0.5 μg/ml ATc; Figure 4D) concentrations. In PP2M419, a steady increase in GFP and RFP levels was observed with increasing concentrations of inducers ATc and arabinose, respectively. In the experimental setup, the concentration of the opposite inducer was kept constant, and the presence of respective non‐cognate inducer did not influence gene expression since inducible expression of GFP and RFP in the same strain were entirely independent of one another.

### Production of indigoidine in M2

**FIGURE 5:**
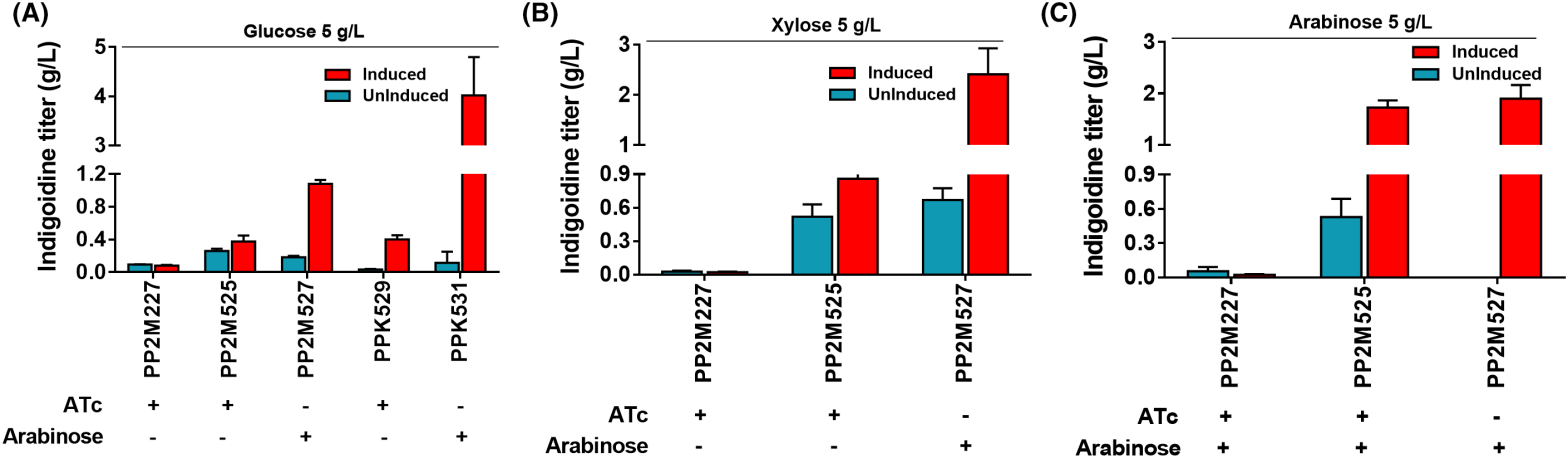
Indigoidine production in M2 (PP2M227, 525, 527) and KT2440 (PPK529, 531) in minimal medium with different C‐sources namely, glucose (A), xylose (B) and arabinose (C). The strain harbouring an empty vector (PP2M227) was used as negative control and uninduced counterparts for determining background fluorescence. The recombinant strains expressed plasmid‐encoded heterologous genes *bpsA* from *S. lavendulae* and *sfp* from *B. subtilis* for conversion of glutamine to indigoidine under the control of either ATc‐inducible (P_*tetR/tetA*_) or arabinose‐inducible (P_*bad*_) promoters. The samples were taken for each strain directly from 30 ml culture tubes with 5 ml of media at the late exponential phase (~16 h) to measure absorbance at OD_612nm_. The inducers were added at *t* = 0 h. the (+) sign below each strain corresponds to inducer added to induce gene expression and (−) sign corresponds to absence of inducer. For strain description, refer to Table 1. Data represent mean values of triplicate assays from at least two individual cultivations, and error bars represent standard deviations.

The production of blue pigment indigoidine has been demonstrated in *P. putida* KT2440 from glucose and from xylose in an engineered strain (Eng et al., 2021; Lim et al., 2021). Since strain M2 natively grows on xylose and arabinose, the ability of this strain to produce indigoidine from C6 and C5 sugars was tested. Two heterologous genes, *bpsA* from *Streptomyces lavendulae* that encodes blue pigment synthetase A and *sfp* from *Bacillus subtilis* that encode 4′‐phosphopantetheinyl transferase were cloned into pRGPDuo vectors under the control of P_*teR/tetA*_ or P_*bad*_. The plasmids were electroporated in M2 and KT2440 to generate recombinant strains for M2 (PP2M227, 525, 527) and KT2440 (PPK529, 531) (Table 1; Table S1). For indigoidine quantification, the strain PP2M227 harbouring an empty vector was used as a negative control, and the respective uninduced counterparts were used to determine the basal production of indigoidine. The recombinant strains were cultured in 30 ml tubes with 5 ml minimal media supplemented with 5 g/L (0.5% w/v) of either hexose (glucose) or pentose (xylose, arabinose) sugars as the carbon source for production experiments. The protein production was induced by adding the respective inducer (at *t* = 0 h, OD_600_ = 0.1). In most of the test strains, the production of blue‐coloured pigment was quite apparent in culture tubes after induction (Figure S4). As expected, the negative control strains PP2M227 did not produce indigoidine. The indigoidine titres varied from the lowest 0.4 ± 0.08 g/L in PP2M525 to the highest 3.68 ± 0.23 g/L in PPK531 for glucose, the lowest 0.9 ± 0.08 g/L in PP2M525 to the highest 2.41 ± 0.5 g/L in PP2M527 for xylose, and the lowest 1.56 ± 0.05 g/L in PP2M525 to the highest 1.89 ± 0.2 g/L in PP2M527 for arabinose (Figure 5A–C). The observation of indigoidine production in the absence of an inducer was consistent with background levels of expression from the P_*tetR/tetA*_ and P_*bad*_ promoters with GFP.

### CRAGE‐mediated gene insertion in the chromosome of environmental isolate M2

Chromosomal gene integration is a complement to the plasmid‐based expression of heterologous genes and pathways. Chassis‐independent recombinase‐assisted genome engineering (CRAGE) technology has recently been developed, which allows single‐step integration of large, complex synthetic constructs into chromosomes of diverse bacterial species with great accuracy and efficiency (Wang et al., 2019). The method involves the sequential use of two plasmids, pW17 (integration of landing pad) and pW34 (integration of target gene). In the first step, the transformation of pW17 leads to the integration of landing pad (LP) comprising a *cre* recombinase gene from phage P1 flanked by mutually exclusive *loxP* sites. The second construct (pW34) is an accessory vector that allows cloning of heterologous genes of interest flanked by the same *lox* sites. On conjugation with the recipient strain, Cre recombinase mediates the insertion of target genes into the LP under the control of the IPTG‐inducible T7 promoter (P_*T7*_).

To expand the toolkit for genetic modification strategies in strain M2, the amenability of this strain to CRAGE‐mediated gene integration was tested. Strain KT2440 was used as a comparison, as the CRAGE method has been previously demonstrated in KT2440 (Wang et al., 2019). First, the vector pW17 was introduced into recipient M2 (and KT2440) via conjugation to integrate the LP into the chromosome of recipient strain. Electroporation of the CRAGE plasmids (pW17 and pW34) proved to be unsuccessful in M2, but electroporation in KT2440 was successful although it proceeded at low efficiency. To maintain consistency, conjugation in both hosts for CRAGE based experiments was used for transformation. The chromosomal integration of LP in M2 and KT2440 was confirmed using colony PCR followed by the growth of strains in the presence of kanamycin selection marker. PacBio sequencing identified pW17 integration at locus tag PP_3007, which encodes a hypothetical protein in KT2440 (PPK706). PacBio sequencing results for M2 (PP2M729) identified multiple sequences and some of the sequences aligned to pW17 confirming the integration of LP into chromosome but the exact genome location could not be identified. Nevertheless, growth of strains in presence of antibiotic (kanamycin) and colony PCR confirmed the chromosomal integration of LP in M2.

**FIGURE 6:**
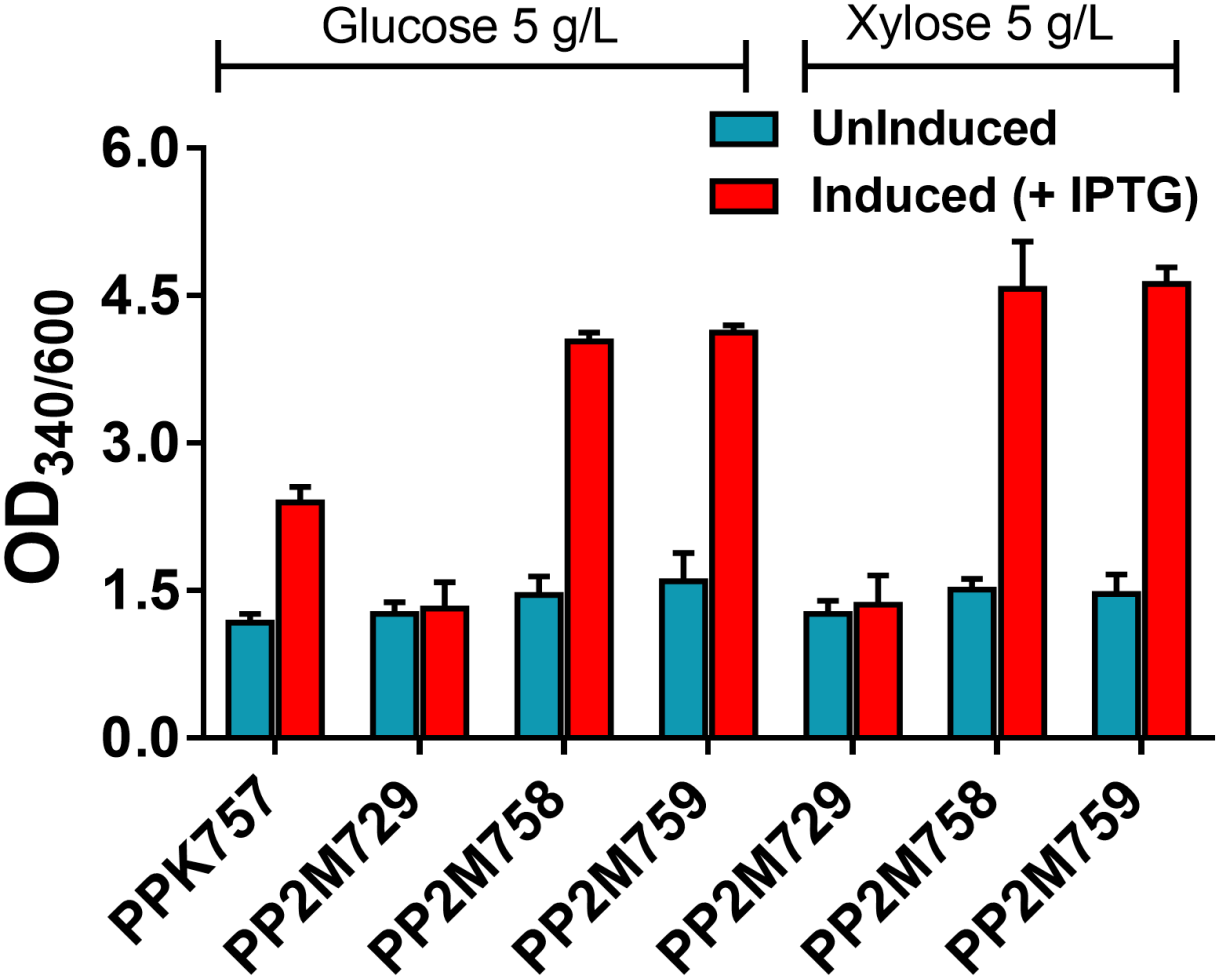
Application of CRAGE technology for production of non‐native bioproduct flaviolin. The red‐coloured flaviolin pigment was quantified in M2 in a minimal medium with glucose and xylose. The graphs consist of relative OD_340nm_ normalized to OD_600_ at the late exponential phase in induced and uninduced states. RppA protein from *S. coelicolor* catalyses malonyl‐CoA conversion to THN that spontaneously oxidizes to red‐coloured pigment flaviolin (Figure S6). Genes coding for rppA‐NT (*S. coelicolor rppA* gene with C‐terminal truncation of 25 amino acids) was cloned into accessory vector pW34 and conjugated to both KT2440 and M2 containing LP to insert *rppA* into the genome of the respective clone. For strain description, refer to Table 1. Data represent mean values of triplicate assays from at least two individual cultivations, and error bars represent standard deviations.

To demonstrate the robustness of CRAGE technology for genome engineering in M2, we evaluated the production of another non‐native pigment molecule flaviolin by integrating *rppA* gene, which codes for 1,3,6,8‐tetrahydroxynaphthalene synthase (RppA). This protein was previously repurposed to use as a malonyl‐CoA biosensor in *P. putida* (Incha et al., 2020; Yang et al., 2018). RppA catalyses the conversion of malonyl‐CoA to 1,3,6,8‐tetrahydroxynaphthalene (THN), which spontaneously oxidizes to a red‐coloured product flaviolin. Flaviolin can be easily quantified by measuring absorbance at 340 nm. The plasmid pW34‐*rppA*‐NT harbouring *rppA* gene controlled by IPTG‐inducible promoter (P_*T7*_) was conjugated into LP‐harbouring M2 strains to generate RppA expressing isolates, namely, PP2M758–759 (M2; #2, 4); the conjugation was successful for two of the four clones harbouring the LP. To confirm the chromosomal integration of *rppA* gene, flaviolin production was measured. The recombinant strains were analysed for flaviolin production (dense red colour intensity) and quantification in 5 ml cultures for endpoint absorbance measurement (OD_340nm_ and OD_600nm_) at late exponential phase (at ~16 h after induction) in glucose and xylose (Figure 6). Continuous absorbance (A_340nm_) measurements on plate reader for 48 h also indicated an increase in flaviolin production in test strains (Figure S5). For M2, the strain PP2M729 harbouring landing pad was used as negative control and the KT2440 strain PPK757 with genomic integration of *rppA* as positive control. The respective uninduced counterpart was used to determine the basal expression of flaviolin in the absence of an inducer (IPTG). In all CRAGE constructs tested, there was an increase in the production of flaviolin in cultures on induction (Figure 6). Production of flaviolin in the absence of the inducer for the test strains (PP2M758–759) was comparable to the control strain (PP2M729) and titres were in the similar range indicating tight gene regulation in CRAGE constructs. The production of red‐coloured pigment was quite noticeable by naked eyes in culture tubes on induction, whereas flaviolin production was very low to negligible in the absence of IPTG (Figure S6). This result again corroborates the tighter control over gene expression in CRAGE constructs.

## DISCUSSION

*Pseudomonads* are globally employed for bioremediation due to their impressive capability to degrade a wide variety of persistent toxic compounds (Mishra et al., 2021). This broad metabolic capability has recently been exploited to adapt them as an excellent host for production of value‐added compounds, such as biofuels and bioproducts. Different strains of *P. putida* exhibit unique stress response mechanisms that could not be produced using model hosts such as *E. coli* (Bitzenhofer et al., 2021). The HV1 certified *P. putida* KT2440 has emerged as the most intensively studied bioproduction host (Kampers et al., 2019). Despite numerous advantages, KT2440 lacks high solvent tolerance for bioconversion of toxic organic compounds and cannot metabolize certain sugars (e.g. xylose, arabinose, galactose) present in lignocellulosic biomass. To enable complete lignocellulose consumption, non‐native catabolic pathways were incorporated in *Pseudomonas* strains, but several limitations remain such as slower growth and non‐ideal phenotypes (Elmore et al., 2020). The solvent tolerance of other *P. putida* group strains: S12, DOT‐T1E and *P. taiwanensis* VLB120 make them promising candidates for conversion of toxic substrates compared to the KT2440 strain (Bretschneider et al., 2022; Rühl et al., 2009). *P. taiwanensis* VLB120 also has a xylose utilization pathway that has been characterized, but it does not grow on *p*‐coumarate and L‐arabinose, limiting its applications for growth on biomass hydrolysate (Wordofa & Kristensen, 2018). *Pseudomonas putida* M2 was recently isolated and possesses native pathways to metabolize a wide range of biomass‐derived carbon sources, including pentose sugars (xylose and arabinose) and aromatics (*p*‐coumarate) (Park, Fong, et al., 2022). Developing genetic tools that are portable to multiple *Pseudomonas* strains beyond KT2440 would allow access to the most promising capabilities found in other strains.

We have demonstrated that genetic tools for plasmid‐based and chromosomal protein expression that were successfully deployed in strain KT2440 could be translated to strain M2 to begin to create a versatile set of tools for this potential new host. These tools enabled production of indigoidine, a non‐ribosomal peptide, by plasmid‐based expression and flaviolin, a polyketide, by chromosomal expression. Among the recombinant strains tested here, KT2440 produced the highest indigoidine titres. While the indigoidine titres were comparatively lower in M2, it is important to acknowledge that KT2440 is a well‐characterized host in terms of heterologous production, and this is the first report for heterologous production in M2. Moreover, differences in product titre among KT2440 and M2 likely reflect the range of differences in distinct hosts. Compared to previous studies that aimed to demonstrate production of valuable products such as indigoidine (Lim et al., 2021), rhamnolipids and pyocyanin (Bator et al., 2020) on xylose with engineered or adapted model *P. putida* strain KT2440, we demonstrated the production of non‐native products on xylose with a native *P. putida* host. However, multiple factors need to be taken into account for process optimization, including: (i) stoichiometries of the pathway for flux balance analysis (Bator et al., 2020), (ii) pathway protein abundance due to changes in promoter expression in levels (Wang et al., 2018), (iii) investigating toxicity and saturation of targeted product (Hernandez‐Arranz et al., 2019), (iv) plasmid stability, and (v) different promoter system (besides arabinose‐inducible P_*bad*_) that does not contribute towards cell growth (Park, Gauttam, et al., 2022). In a parallel study, the amenability of this strain to CRISPR interference (CRISPRi) technology was established to identify gene products involved in pentose sugar oxidation and to identify conditionally essential genes for xylose oxidation (Park, Gauttam, et al., 2022). Recently, other *Pseudomonas* strains have been engineered to be more effective hosts for bioconversion. The genome of *P. taiwanensis* VLB120 has been streamlined by targeted genome reduction (Wynands et al., 2019) and protein expression in VLB120 has been controlled by manipulating mRNA stability (Neves et al., 2020). For *P. putida* S12, CRISPR and λ‐Red recombineering were used to integrate genes for 2,5‐furandicarboxylic acid production (Pham et al., 2020). These studies and the work described here demonstrate the promise of developing versatile genetic tools that can be applied to the *Pseudomonads*. The ability to translate tools rapidly from the well‐studied strain KT2440 to environmental isolates may be a catalyst to perform targeted isolation of *Pseudomonas* species with defined properties that are favourable for bioconversion.

## AUTHOR CONTRIBUTIONS

**Rahul Gauttam:** Conceptualization (lead); data curation (lead); formal analysis (lead); visualization (lead); writing – original draft (lead); writing – review and editing (lead). **Thomas Eng:** Investigation (supporting); methodology (supporting). **Zhiying Zhao:** Data curation (supporting); formal analysis (supporting); methodology (supporting); writing – review and editing (supporting). **Qurrat ul ain Rana:** Investigation (supporting); methodology (supporting). **Blake A. Simmons:** Funding acquisition (supporting); project administration (supporting); supervision (supporting); writing – review and editing (supporting). **Yasuo Yoshikuni:** Funding acquisition (supporting); methodology (supporting); supervision (supporting). **Aindrila Mukhopadhyay:** Funding acquisition (supporting); project administration (supporting); supervision (supporting). **Steven W. Singer:** Conceptualization (lead); funding acquisition (lead); investigation (lead); project administration (lead); supervision (lead); writing – original draft (lead); writing – review and editing (lead).

## FUNDING INFORMATION

U.S. Department of Energy (Grant / Award Number: DE‐AC02‐05CH11231).

## CONFLICT OF INTEREST

The authors declare that they have no conflicts of interest.

## ETHICAL APPROVAL

Not applicable.

## Supporting information


Appendix S1.
