# Supplementary material for: Development of genetic tools for heterologous protein expression in a pentose‐utilizing environmental isolate of Pseudomonas putida
Source: Microb Biotechnol. 2023 Jan 24;16(3):645–61. doi: 10.1111/1751-7915.14205 (PMC9948227; doi:10.1111/1751-7915.14205)
Supplement: Supplementary file 1 — Appendix S1. [file MBT2-16-645-s001.docx]

**Development of genetic tools for heterologous protein expression in pentose-utilizing environmental isolate of *Pseudomonas putida***

Rahul Gauttam^1,2^, Thomas Eng^1,2^, Zhiying Zhao^3,4^, , Qurrat ul ain Rana^1^, Aindrila Mukhopadhyay^1,2^, Blake A. Simmons ^1,2^, Yasuo Yoshikuni^3,4^, Steven W. Singer^1,2^

^1^*The Joint BioEnergy Institute, Emeryville, CA, USA*

*^2^Biological Systems and Engineering Division, Lawrence Berkeley National Laboratory, Berkeley, CA, USA*

*^3^Joint Genome Institute, Berkeley, CA, USA*

*^4^Environmental Genomics and Systems Biology Division, Lawrence Berkeley National Laboratory, Berkeley, CA, USA*

* Corresponding author. Mailing address: Lawrence Berkeley National Laboratory, 1 Cyclotron Road, Berkeley, CA, USA, 94720. Phone: +1 510-486-5556. E-mail: swsinger@lbl.gov

**Supplementary methods**

**Construction of pRGPDuo6, pRGPDuo7 and pRGPDuo8-derived vectors with RBS0, RBS8 and RBS10:** The gene encoding for sfGFP with RBS (RBS0, 8, 10) was amplified using pRGPDuo4-sfGFPbad (Gauttam et al., 2021) and respective primer set depending on RBS sequence incorporated in oligonucleotides. Refer Table S2 for primer sequence pair used for a particular plasmid. The plasmids pRGPDuo6-sfGFPcymR RBS0, 8, 10 were constructed by ligating BglII/NheI-digested pRGPDuo6 with BglII/NheI-digested PCR product. Similarly, plasmids pRGPDuo7-sfGFPrhaRS RBS0, 8, 10 and plasmids pRGPDuo8-sfGFPcymR RBS0, 8, 10 were constructed by ligating PstI/KpnI-digested pRGPDuo7 and pRGPDuo8 with PstI/KpnI-digested PCR product from template pRGPDuo4-sfGFPbad.

**Construction of pRGPDuo2-derived vectors with eleven different RBSs 1-11 controlled by IPTG inducible P*_tac_*:** The gene encoding for sfGFP with specific RBS (RBS1-11) was amplified using pRGPDuo4-sfGFPtac (Gauttam et al., 2021) and respective primer set depending on the RBS sequence incorporated in oligonucleotides. Refer Table S2 for primer sequence pair used for a particular plasmid. 11 plasmids pRGPDuo2-sfGFPtac (RBS1-11) were constructed by ligating PstI/BamHI-digested pRGPDuo2 with PstI/BamHI-digested PCR product.

**Construction of pRGPDuo2-derived vectors with eleven different RBSs 1-11 controlled by ATc inducible P*_tetR/tetA_*:** The gene encoding for sfGFP with specific RBS (RBS1-11) was amplified using pRGPDuo4-sfGFPbad (Gauttam et al., 2021) and respective primer set depending on the RBS sequence incorporated in oligonucleotides. Refer Table S2 for primer sequence pair used for a particular plasmid. 11 plasmids pRGPDuo2-sfGFPtet (RBS1-11) were constructed by ligating BglII/NheI-digested pRGPDuo2 with BglII/NheI-digested PCR product.

**Construction of pRGPDuo4-derived vectors with eleven different RBSs 1-11 controlled by arabinose inducible P*_bad_*:** The gene encoding for sfGFP with specific RBS (RBS1-11) was amplified using pRGPDuo4-sfGFPbad (Gauttam et al., 2021) and respective primer set depending on the RBS sequence incorporated in oligonucleotides. Refer Table S2 for primer sequence pair used for a particular plasmid. 11 plasmids pRGPDuo4-sfGFPbad (RBS1-11) were constructed by ligating BglII/NheI-digested pRGPDuo4 with BglII/NheI-digested PCR product.

**Construction of pW34-rppA NT:** The gene encoding for *rppA* from *S. coelicolor* was amplified using pBADT-*rppA*-NT (Incha et al., 2020) as template and primers 101/102CRAGErppA NT fwd/rev. The NdeI/NheI-digested PCR product was ligated into NdeI/NheI-digested pW34 to create CRAGE accessory vector pW34-rppA NT. The pW34-rppA NT was transformed into four clones of each isolate harbouring landing pad pW17.

**Construction of indigoidine plasmids pRGPDuo2-sfpbpsAtet and pRGPDuo4-sfpbpsAbad:** To construct the plasmid pRGPDuo2-sfpbpsAtet, the BglII/AvrII-digested fragment from pTE251 that contained the nucleotide sequence for target genes responsible for indigoidine production (*spf* and *bpsA*) was ligated into BglII/NheI-digested pRGPDuo2 under the control of P*_tetR/tetA._* To construct the plasmid pRGPDuo4-sfpbpsAbad, the BglII/NotI-digested fragment from pTE251 that contained the nucleotide sequence for target genes responsible for indigoidine production (*spf* and *bpsA*) was ligated into BglII/NotI-digested pRGPDuo4 under the control of P*_bad._*

**Supplementary Tables**

**Table S1:** Expression plasmids used in this study.

| **Plasmid(*)** | **Relevant characteristics** | **Source / Reference** |
| --- | --- | --- |
| pRGPDuo1 | dual-inducible *E. coli/P. putida* shuttle vector (*P_tac_*, *lacI^Q^,* OriV*_P. putida_* (pBBR1), OriV*_E. coli_* (pBBR1), *P_tetR/tetA_, tetR*); Gent^R^ | Gauttam *et al*., 2021 |
| pRGPDuo2 | dual-inducible *E. coli/P. putida* shuttle vector (*P_tac_*, *lacI^Q^,* OriV*_P. putida_* (pRO1600), OriV*_E. coli_* (colE1), *P_tetR/tetA_, tetR*); Kan^R^ | Gauttam *et al*., 2020 |
| pRGPDuo3 | dual-inducible *E. coli/P. putida* shuttle vector (*P_tac_*, *lacI^Q^,* OriV*_P. putida_* (pBBR1), OriV*_E. coli_* (pBBR1), *P_bad_, araC*); Gent^R^ | Gauttam *et al*., 2021 |
| pRGPDuo4 | dual-inducible *E. coli/P. putida* shuttle vector (*P_tac_*, *lacI^Q^,* OriV*_P. putida_* (pRO1600), OriV*_E. coli_* (colE1), *P_bad_, araC*); Kan^R^ | Gauttam *et al*., 2021 |
| pRGPDuo6 | dual-inducible *E. coli/P. putida* shuttle vector (*P_tac_*, *lacI^Q^,* OriV*_P. putida_* (pRO1600), OriV*_E. coli_* (colE1), *P_Cuo_, cymR*); Kan^R^ | This study |
| pRGPDuo7 | dual-inducible *E. coli/P. putida* shuttle vector (*P_bad_*, *araC,* OriV*_P. putida_* (pRO1600), OriV*_E. coli_* (colE1), *P_rhaRS_, rhaRS*); Kan^R^ | This study |
| pRGPDuo8 | dual-inducible *E. coli/P. putida* shuttle vector (*P_bad_*, *araC,* OriV*_P. putida_* (pRO1600), OriV*_E. coli_* (colE1), *P_Cuo_, cymR*); Kan^R^ | This study |
| pJeM1 | Rhamnose-inducible expression vector for replication in *E. coli* and *P. putida* | Jeske and Altenbuchner, 2010 (Addgene) |
| pCT5-bac2.0 | Cumate-inducible expression of sfGFP gene in *Bacillus subtilis*, *B. megaterium* and *E. coli* | Seo and Schmidt- Dannert, 2019 (Addgene) |
| pRGPDuo1-sfGFPtac RBS0 | pRGPDuo1 carrying the gene for super folder green fluorescent protein (sfGFP) under the control of *P_tac_* with RBS0; Gent^R^ | Gauttam *et al*., 2021 |
| pRGPDuo1-sfGFPtet | pRGPDuo1 carrying the gene for super folder green fluorescent protein (sfGFP) under the control of *P_tetR/tetA_* with RBS0; Gent^R^ | Gauttam *et al*., 2021 |
| pRGPDuo2-RFPtet | pRGPDuo2 carrying the gene for red fluorescent protein (RFP) under the control of *P_tetR/tetA_* with RBS0; Kan^R^ | Gauttam *et al*., 2020 |
| pRGPDuo2-sfGFPtac RBS0 | pRGPDuo2 carrying the gene for super folder green fluorescent protein (sfGFP) under the control of *P_tac_* with RBS0; Kan^R^ | Gauttam *et al*., 2020 |
| pRGPDuo2-sfGFPtac RBS1 | pRGPDuo2 carrying the gene for super folder green fluorescent protein (sfGFP) under the control of *P_tac_* with RBS1; Kan^R^ | This study |
| pRGPDuo2-sfGFPtac RBS2 | pRGPDuo2 carrying the gene for super folder green fluorescent protein (sfGFP) under the control of *P_tac_* with RBS2; Kan^R^ | This study |
| pRGPDuo2-sfGFPtac RBS3 | pRGPDuo2 carrying the gene for super folder green fluorescent protein (sfGFP) under the control of *P_tac_* with RBS3; Kan^R^ | This study |
| pRGPDuo2-sfGFPtac RBS4 | pRGPDuo2 carrying the gene for super folder green fluorescent protein (sfGFP) under the control of *P_tac_* with RBS4; Kan^R^ | This study |
| pRGPDuo2-sfGFPtac RBS5 | pRGPDuo2 carrying the gene for super folder green fluorescent protein (sfGFP) under the control of *P_tac_* with RBS5; Kan^R^ | This study |
| pRGPDuo2-sfGFPtac RBS6 | pRGPDuo2 carrying the gene for super folder green fluorescent protein (sfGFP) under the control of *P_tac_* with RBS6; Kan^R^ | This study |
| pRGPDuo2-sfGFPtac RBS7 | pRGPDuo2 carrying the gene for super folder green fluorescent protein (sfGFP) under the control of *P_tac_* with RBS7; Kan^R^ | This study |
| pRGPDuo2-sfGFPtac RBS8 | pRGPDuo2 carrying the gene for super folder green fluorescent protein (sfGFP) under the control of *P_tac_* with RBS8; Kan^R^ | This study |
| pRGPDuo2-sfGFPtac RBS9 | pRGPDuo2 carrying the gene for super folder green fluorescent protein (sfGFP) under the control of *P_tac_* with RBS9; Kan^R^ | This study |
| pRGPDuo2-sfGFPtac RBS10 | pRGPDuo2 carrying the gene for super folder green fluorescent protein (sfGFP) under the control of *P_tac_* with RBS10; Kan^R^ | This study |
| pRGPDuo2-sfGFPtac RBS11 | pRGPDuo2 carrying the gene for super folder green fluorescent protein (sfGFP) under the control of *P_tac_* with RBS11; Kan^R^ | This study |
| pRGPDuo2-sfGFPtet RBS0 | pRGPDuo2 carrying the gene for super folder green fluorescent protein (sfGFP) under the control of *P_tetR/tetA_* with RBS0; Kan^R^ | Gauttam *et al*., 2020 |
| pRGPDuo2-sfGFPtet RBS1 | pRGPDuo2 carrying the gene for super folder green fluorescent protein (sfGFP) under the control of *P_tetR/tetA_* with RBS1; Kan^R^ | This study |
| pRGPDuo2-sfGFPtet RBS2 | pRGPDuo2 carrying the gene for super folder green fluorescent protein (sfGFP) under the control of *P_tetR/tetA_* with RBS2; Kan^R^ | This study |
| pRGPDuo2-sfGFPtet RBS3 | pRGPDuo2 carrying the gene for super folder green fluorescent protein (sfGFP) under the control of *P_tetR/tetA_* with RBS3; Kan^R^ | This study |
| pRGPDuo2-sfGFPtet RBS4 | pRGPDuo2 carrying the gene for super folder green fluorescent protein (sfGFP) under the control of *P_tetR/tetA_* with RBS4; Kan^R^ | This study |
| pRGPDuo2-sfGFPtet RBS5 | pRGPDuo2 carrying the gene for super folder green fluorescent protein (sfGFP) under the control of *P_tetR/tetA_* with RBS5; Kan^R^ | This study |
| pRGPDuo2-sfGFPtet RBS6 | pRGPDuo2 carrying the gene for super folder green fluorescent protein (sfGFP) under the control of *P_tetR/tetA_* with RBS6; Kan^R^ | This study |
| pRGPDuo2-sfGFPtet RBS7 | pRGPDuo2 carrying the gene for super folder green fluorescent protein (sfGFP) under the control of *P_tetR/tetA_* with RBS7; Kan^R^ | This study |
| pRGPDuo2-sfGFPtet RBS8 | pRGPDuo2 carrying the gene for super folder green fluorescent protein (sfGFP) under the control of *P_tetR/tetA_* with RBS8; Kan^R^ | This study |
| pRGPDuo2-sfGFPtet RBS9 | pRGPDuo2 carrying the gene for super folder green fluorescent protein (sfGFP) under the control of *P_tetR/tetA_* with RBS9; Kan^R^ | This study |
| pRGPDuo2-sfGFPtet RBS10 | pRGPDuo2 carrying the gene for super folder green fluorescent protein (sfGFP) under the control of *P_tetR/tetA_* with RBS10; Kan^R^ | This study |
| pRGPDuo2-sfGFPtet RBS11 | pRGPDuo2 carrying the gene for super folder green fluorescent protein (sfGFP) under the control of *P_tetR/tetA_* with RBS11; Kan^R^ | This study |
| pRGPDuo3-sfGFPbad RBS0 | pRGPDuo3 carrying the gene for super folder green fluorescent protein (sfGFP) under the control of *P_bad_* with RBS0; Gent^R^ | Gauttam *et al*., 2021 |
| pRGPDuo4-RFPtac | pRGPDuo4 carrying the gene for red fluorescent protein (RFP) under the control of *P_tac_* with RBS0; Kan^R^ | Gauttam *et al*., 2021 |
| pRGPDuo4-RFPbad | pRGPDuo4 carrying the gene for red fluorescent protein (RFP) under the control of *P_bad_* with RBS0; Kan^R^ | Gauttam *et al*., 2021 |
| pRGPDuo4-RFPtac + sfGFPbad RBS0 | pRGPDuo4 carrying the gene for red fluorescent protein (RFP) under the control of *P_tac_* and super folder green fluorescent protein (sfGFP) under the control of *P_bad_* with RBS0; Kan^R^ | This study |
| pRGPDuo4-sfGFPbad RBS0 | pRGPDuo4 carrying the gene for super folder green fluorescent protein (sfGFP) under the control of *P_bad_* with RBS0; Kan^R^ | Gauttam *et al*., 2021 |
| pRGPDuo4-sfGFPbad RBS1 | pRGPDuo4 carrying the gene for super folder green fluorescent protein (sfGFP) under the control of *P_bad_* with RBS1; Kan^R^ | This study |
| pRGPDuo4-sfGFPbad RBS2 | pRGPDuo4 carrying the gene for super folder green fluorescent protein (sfGFP) under the control of *P_bad_* with RBS2; Kan^R^ | This study |
| pRGPDuo4-sfGFPbad RBS3 | pRGPDuo4 carrying the gene for super folder green fluorescent protein (sfGFP) under the control of *P_bad_* with RBS3; Kan^R^ | This study |
| pRGPDuo4-sfGFPbad RBS4 | pRGPDuo4 carrying the gene for super folder green fluorescent protein (sfGFP) under the control of *P_bad_* with RBS4; Kan^R^ | This study |
| pRGPDuo4-sfGFPbad RBS5 | pRGPDuo4 carrying the gene for super folder green fluorescent protein (sfGFP) under the control of *P_bad_* with RBS5; Kan^R^ | This study |
| pRGPDuo4-sfGFPbad RBS6 | pRGPDuo4 carrying the gene for super folder green fluorescent protein (sfGFP) under the control of *P_bad_* with RBS6; Kan^R^ | This study |
| pRGPDuo4-sfGFPbad RBS7 | pRGPDuo4 carrying the gene for super folder green fluorescent protein (sfGFP) under the control of *P_bad_* with RBS7; Kan^R^ | This study |
| pRGPDuo4-sfGFPbad RBS8 | pRGPDuo4 carrying the gene for super folder green fluorescent protein (sfGFP) under the control of *P_bad_* with RBS8; Kan^R^ | This study |
| pRGPDuo4-sfGFPbad RBS9 | pRGPDuo4 carrying the gene for super folder green fluorescent protein (sfGFP) under the control of *P_bad_* with RBS9; Kan^R^ | This study |
| pRGPDuo4-sfGFPbad RBS10 | pRGPDuo4 carrying the gene for super folder green fluorescent protein (sfGFP) under the control of *P_bad_* with RBS10; Kan^R^ | This study |
| pRGPDuo4-sfGFPbad RBS11 | pRGPDuo4 carrying the gene for super folder green fluorescent protein (sfGFP) under the control of *P_bad_* with RBS11; Kan^R^ | This study |
| pRGPDuo6-sfGFPcymR RBS0 | pRGPDuo6 carrying the gene for super folder green fluorescent protein (sfGFP) under the control of *P_Cuo_* with RBS0; Kan^R^ | This study |
| pRGPDuo6-sfGFPcymR RBS8 | pRGPDuo6 carrying the gene for super folder green fluorescent protein (sfGFP) under the control of *P_Cuo_* with RBS8; Kan^R^ | This study |
| pRGPDuo6-sfGFPcymR RBS10 | pRGPDuo6 carrying the gene for super folder green fluorescent protein (sfGFP) under the control of *P_Cuo_* with RBS10; Kan^R^ | This study |
| pRGPDuo7-sfGFPrhaRS RBS0 | pRGPDuo7 carrying the gene for super folder green fluorescent protein (sfGFP) under the control of *P_rhaRS_* with RBS0; Kan^R^ | This study |
| pRGPDuo7-sfGFPrhaRS RBS8 | pRGPDuo7 carrying the gene for super folder green fluorescent protein (sfGFP) under the control of *P_rhaRS_* with RBS8; Kan^R^ | This study |
| pRGPDuo7-sfGFPrhaRS RBS10 | pRGPDuo7 carrying the gene for super folder green fluorescent protein (sfGFP) under the control of *P_rhaRS_* with RBS10; Kan^R^ | This study |
| pRGPDuo8-sfGFPcymR RBS0 | pRGPDuo8 carrying the gene for super folder green fluorescent protein (sfGFP) under the control of *P_Cuo_* with RBS0; Kan^R^ | This study |
| pRGPDuo8-sfGFPcymR RBS8 | pRGPDuo8 carrying the gene for super folder green fluorescent protein (sfGFP) under the control of *P_Cuo_* with RBS8; Kan^R^ | This study |
| pRGPDuo8-sfGFPcymR RBS10 | pRGPDuo8 carrying the gene for super folder green fluorescent protein (sfGFP) under the control of *P_Cuo_* with RBS10; Kan^R^ | This study |
| pW17 | Plasmid containing a mariner transposon and transposase. The transposon contained a Cre recombinase gene and a kanamycin-resistant gene flanked by two mutually exclusive lox sites (loxP and lox5171) | Wang et al., 2019 |
| pW34 | The R6Kr based plasmid encoding a BGC under the control of the T7 promoter and an apramycin resistant gene flanked by the two mutually exclusive lox sites. | Wang et al., 2019 |
| pW34-rppA-NT | pW34 with nucleotide sequence for gene encoding for RppA | This study |
| pTE251 | For the gene amplification for the indigoidine production. This plasmid was used as a template for the amplification of *sfp* encoding 4’-phosphopantetheinyl transferase from *Bacillus subtilis* and *bpsA* encoding blue pigment synthetase A from *Streptomyces lavendulae*; Kan^R^ | This study |
| pRGPDuo2-sfpbpsAtet | pRGPDuo2 carrying the gene for *sfp* and *bpsA* under the control of *P_tetR/tetA_* with RBS0; Kan^R^ | This study |
| pRGPDuo3-sfpbpsAbad | pRGPDuo3 carrying the gene for *sfp* and *bpsA* under the control of *P_bad_* with RBS0; Gent^R^ | This study |
| pRGPDuo4-sfpbpsAbad | pRGPDuo4 carrying the gene for *sfp* and *bpsA* under the control of *P_bad_* with RBS0; Kan^R^ | This study |

**Table S2:** Oligonucleotides used in this study for creating expression plasmids. Restriction sites are indicated in bold.

| **Oligonucleotides** | **Sequence (5’ → 3’)** | **Plasmid construction / purpose** |
| --- | --- | --- |
| 55cumateduo2 fwd | aacgagt**gaattc**aaactagttcacactggctcaccttcg | Construction of pRGPDuo6 using pRGPDuo2 |
| 56cumateduo2 rev | aaga**agatct**cttaaggcaattgaccggttaatcataatacaaacagaccagattgtctg |  |
| 57rhaRduo4 fwd | gcctttcaggctgcgcaactgcTATTATACGCAAGGCGAC | Construction of pRGPDuo7 employing Gibson assembly method and using pRGPDuo4 (vector backbone) and pJeM1 as template (for amplification of rhaR and rhaS inserts). |
| 58rhaRduo4 rev | tgcaataacgcGAATCTTCTCAACGTATTTGTAC |  |
| 59rhaSduo4 fwd | tgagaagattcGCGTTATTGCAGAAAGCC |  |
| 60rhaSduo4 rev | gatcctctagagtcgacctgcaggcatgcTAAGAATTGTTCATTACGACCAG |  |
| 61cumateduo4 fwd | gcctttcaggctgcgcaactgcgtacgGTTCACACTGGCTCACCTTC | Construction of pRGPDuo8 employing Gibson assembly and using pRGPDuo2 as vector backbone pCT5-bac2.0 for cymR-Cuo amplification. |
| 62cumateduo4 rev | gatcctctagagtcgacctgcaggcatgcCTTATTAGTTAATCATAATACAAACAGACCA |  |
| DuoRFP_fwd | aacgc**ctgcag**gcttttaagaaggagatatacatatggcgagtagcg | Cloning of RFP in pRGPDuo4 to create pRGPDuo4-RFPtac |
| DuoRFP_rev | gga**ggatcc**ttactcgagtttggatcct |  |
| DuosfGFP-fwd | ctgcagagctgcaggaattc**agatct**gattaaagaggagaaattaagcatgagcaaagg | Cloning of sfGFP in pRGPDuo4-RFPtac to create pRGPDuo4-RFPtac + sfGFPbad RBS0 |
| DuosfGFP-rev | gctagcagct**gctagc**tttggatccttatttgtagagctcatcca |  |
| **Oligonucleotides used for RBS1-11 (and sfGFP) cloning into pRGPDuo2 under P*_tac_* promoter** | | |
| 63duo4RBSs tac rev | acatga**ggatcc**ATCAGACCGCTTCTGCGTT | Common reverse primer for RBS1-11 (and sfGFP) cloning in pRGPDuo2 |
| 64duo4RBS1tac | tacc**ctgcag**gTGACCATCCCCCCCTTAAGCGAGGTATTAAatgagcaaaggagaagaac | pRGPDuo2-sfGFPtac RBS1 |
| 65duo4RBS2tac | tacc**ctgcag**gATCAAATAAAAAAAGGAGGTAGTCACCCTatgagcaaaggagaagaac | pRGPDuo2-sfGFPtac RBS2 |
| 66duo4RBS3tac | acc**ctgcag**gCATAAAGAGCTAGATCCTCGGGAGGTCAAGTatgagcaaaggagaagaac | pRGPDuo2-sfGFPtac RBS3 |
| 67duo4RBS4tac | tacc**ctgcag**gAAAGGTTAAGCACAAAAGAGGAGGAGCACTatgagcaaaggagaagaac | pRGPDuo2-sfGFPtac RBS4 |
| 68duo4RBS5tac | acc**ctgcag**gTGAGGGGACTACACGTCAAAGGAGGTCGAGAatgagcaaaggagaagaac | pRGPDuo2-sfGFPtac RBS5 |
| 69duo4RBS6tac | acc**ctgcag**gAGAATTTTTAGGAGCTAATAGGGAGGTCAGAatgagcaaaggagaagaac | pRGPDuo2-sfGFPtac RBS6 |
| 70duo4RBS7tac | tacc**ctgcag**gAGAAAGAAAAGAGGAACGAGGAGGTATTTAatgagcaaaggagaagaac | pRGPDuo2-sfGFPtac RBS7 |
| 71duo4RBS8tac | acc**ctgcag**GAAAGAATCCTATCCCAATAAGGAGGTATATTatgagcaaaggagaagaac | pRGPDuo2-sfGFPtac RBS8 |
| 72duo4RBS9tac | acc**ctgcag**TCAAATAATATTAAGCGATAAGGAGGTTTTAGatgagcaaaggagaagaac | pRGPDuo2-sfGFPtac RBS9 |
| 73duo4RBS10tac | tacc**ctgcag**gCACAGGAAGTACGGTATAAGGAGGTAGGGAatgagcaaaggagaagaac | pRGPDuo2-sfGFPtac RBS10 |
| 74duo4RBS11tac | acc**ctgcag**AATAATTTTGTTTAACTTTAAGAAGGAGATATAatgagcaaaggagaagaa | pRGPDuo2-sfGFPtac RBS11 |
| **Oligonucleotides used for RBS1-11 (and sfGFP) cloning into pRGPDuo2 under Ptet promoter and into pRGPDuo4 under Pbad promoter** | | |
| 75duo4RBSs bad rev | atgaactagt**gctagc**CTGGACCGCGGTACTCCG | Common reverse primer for RBS1-11 (sfGFP) cloning in pRGPDuo2 and pRGPDuo4 |
| 76duo4RBS1bad | agatt**agatct**TGACCATCCCCCCCTTAAGCGAGGTATTAAatgagcaaaggagaagaac | pRGPDuo2-sfGFPtet RBS1 and  pRGPDuo4-sfGFPbad RBS1 |
| 77duo4RBS2bad | agatt**agatct**ATCAAATAAAAAAAGGAGGTAGTCACCCTatgagcaaaggagaagaact | pRGPDuo2-sfGFPtet RBS2 and  pRGPDuo4-sfGFPbad RBS2 |
| 78duo4RBS3bad | gatt**agatct**CATAAAGAGCTAGATCCTCGGGAGGTCAAGTatgagcaaaggagaagaac | pRGPDuo2-sfGFPtet RBS3 and  pRGPDuo4-sfGFPbad RBS3 |
| 79duo4RBS4bad | agatt**agatct**AAAGGTTAAGCACAAAAGAGGAGGAGCACTatgagcaaaggagaagaac | pRGPDuo2-sfGFPtet RBS4 and  pRGPDuo4-sfGFPbad RBS4 |
| 80duo4RBS5bad | gatt**agatct**TGAGGGGACTACACGTCAAAGGAGGTCGAGAatgagcaaaggagaagaac | pRGPDuo2-sfGFPtet RBS5 and  pRGPDuo4-sfGFPbad RBS5 |
| 81duo4RBS6bad | gatt**agatct**AGAATTTTTAGGAGCTAATAGGGAGGTCAGAatgagcaaaggagaagaac | pRGPDuo2-sfGFPtet RBS6 and  pRGPDuo4-sfGFPbad RBS6 |
| 82duo4RBS7bad | agatt**agatct**AGAAAGAAAAGAGGAACGAGGAGGTATTTAatgagcaaaggagaagaac | pRGPDuo2-sfGFPtet RBS7 and  pRGPDuo4-sfGFPbad RBS7 |
| 83duo4RBS8bad | att**agatct**GAAAGAATCCTATCCCAATAAGGAGGTATATTatgagcaaaggagaagaac | pRGPDuo2-sfGFPtet RBS8 and  pRGPDuo4-sfGFPbad RBS8 |
| 84duo4RBS9bad | att**agatct**TCAAATAATATTAAGCGATAAGGAGGTTTTAGatgagcaaaggagaagaac | pRGPDuo2-sfGFPtet RBS9 and  pRGPDuo4-sfGFPbad RBS9 |
| 85duo4RBS10bad | agatt**agatct**CACAGGAAGTACGGTATAAGGAGGTAGGGAatgagcaaaggagaagaac | pRGPDuo2-sfGFPtet RBS10 and  pRGPDuo4-sfGFPbad RBS10 |
| 86duo4RBS11bad | att**agatct**AATAATTTTGTTTAACTTTAAGAAGGAGATATAatgagcaaaggagaagaa | pRGPDuo2-sfGFPtet RBS11 and  pRGPDuo4-sfGFPbad RBS11 |
| **Oligonucleotides used for RBSs (and sfGFP) cloning into pRGPDuo6 under P*_Cuo_* promoter** | | |
| 91duo6GFP rev | tcagag**gctagc**ggatccttatttgtagagctcatccatg | Common reverse primer for RBSs (sfGFP) cloning in pRGPDuo6 |
| 92duo6GFPRBS0 fwd | agatc**agatct**AAAGATCTGATTAAAGAGGAGAAATTAAGCatgagcaaaggagaagaac | pRGPDuo6-sfGFPcymR RBS0 |
| 93duo6GFPRBS8 fwd | gatc**agatct**GAAAGAATCCTATCCCAATAAGGAGGTATATTatgagcaaaggagaagaa | pRGPDuo6-sfGFPcymR RBS8 |
| 94duo6GFPRBS10 fwd | agatc**agatct**CACAGGAAGTACGGTATAAGGAGGTAGGGAatgagcaaaggagaagaac | pRGPDuo6-sfGFPcymR RBS10 |
| **Oligonucleotides used for RBSs (and sfGFP) cloning into pRGPDuo7 and pRGPDuo8 under P*_rhaRS_* and P*_Cuo_* promoters respectively** | | |
| 95duo78GFP rev | tcagag**ggtacc**ggatccttatttgtagagctcatccatg | Common reverse primer for RBSs (sfGFP) cloning in pRGPDuo7 and pRGPDuo8 |
| 96duo78GFPRBS0 | agatc**ctgcag**AAAGATCTGATTAAAGAGGAGAAATTAAGCatgagcaaaggagaagaac | pRGPDuo7-sfGFPrhaRS RBS0 and  pRGPDuo8-sfGFPcymR RBS0 |
| 97duo78GFPRBS08 | gatc**ctgcag**GAAAGAATCCTATCCCAATAAGGAGGTATATTatgagcaaaggagaagaa | pRGPDuo7-sfGFPrhaRS RBS8 and  pRGPDuo8-sfGFPcymR RBS8 |
| 98duo78GFPRBS10 fwd | agatc**ctgcag**CACAGGAAGTACGGTATAAGGAGGTAGGGAatgagcaaaggagaagaac | pRGPDuo7-sfGFPrhaRS RBS10 &  pRGPDuo8-sfGFPcymR RBS10 |
| LPdetection_fwd | gaccgagatagggttgagtgttgttcc | Colony PCR for the detection of Landing pad |
| LPdetection_rev | aaccttcgtgtagacttccgttgaactgat |  |
| pW17detection_fwd | ccaccttcgtaagactgtagtg | Colony PCR for detection of pW17 |
| pW17detection_rev | tcccagatctcaaactggaacaacactc |  |
| 99CRAGEGFP fwd | ag**catatg**tagctagtcatatgagcaaaggagaagaacttttcact | Cloning of sfGFP in the plasmid pW34 to create pW34-sfGFP for CRAGE mediated recombineering. |
| 100CRAGEGFP rev | acgtgaattc**gctagc**ttatttgtagagctcatccatgccatg |  |
| 101CRAGErppA NT fwd | gaaggagatatacatatggcgactttgtgcagacc | Cloning of *rppA* NT in the plasmid pW34 to create pW34-rppA NT for CRAGE mediated recombineering. |
| 102CRAGErppA NT rev | cctagggctagcgtataaacgcagaaaggccca |  |

**Table S3:** RBSs used in this study from literature (Wang et al., 2018).

| **RBS name** | **RBS sequence** |  |
| --- | --- | --- |
| RBS0 | ^AAAGAGGAGAA^ | |
| RBS1 | ^TGACCATCCCCCCCTTAAGCGAGGTATTAA^ | |
| RBS2 | ^ATCAAATAAAAAAAGGAGGTAGTCACCCT^ | |
| RBS3 | ^CATAAAGAGCTAGATCCTCGGGAGGTCAAGT^ | |
| RBS4 | ^AAAGGTTAAGCACAAAAGAGGAGGAGCACT^ | |
| RBS5 | ^TGAGGGGACTACACGTCAAAGGAGGTCGAGA^ | |
| RBS6 | ^AGAATTTTTAGGAGCTAATAGGGAGGTCAGA^ | |
| RBS7 | ^AGAAAGAAAAGAGGAACGAGGAGGTATTTA^ | |
| RBS8 | ^GAAAGAATCCTATCCCAATAAGGAGGTATATT^ | |
| RBS9 | ^TCAAATAATATTAAGCGATAAGGAGGTTTTAG^ | |
| RBS10 | ^CACAGGAAGTACGGTATAAGGAGGTAGGGA^ | |
| RBS11 | ^AATAATTTTGTTTAACTTTAAGAAGGAGATATA^ | |

**
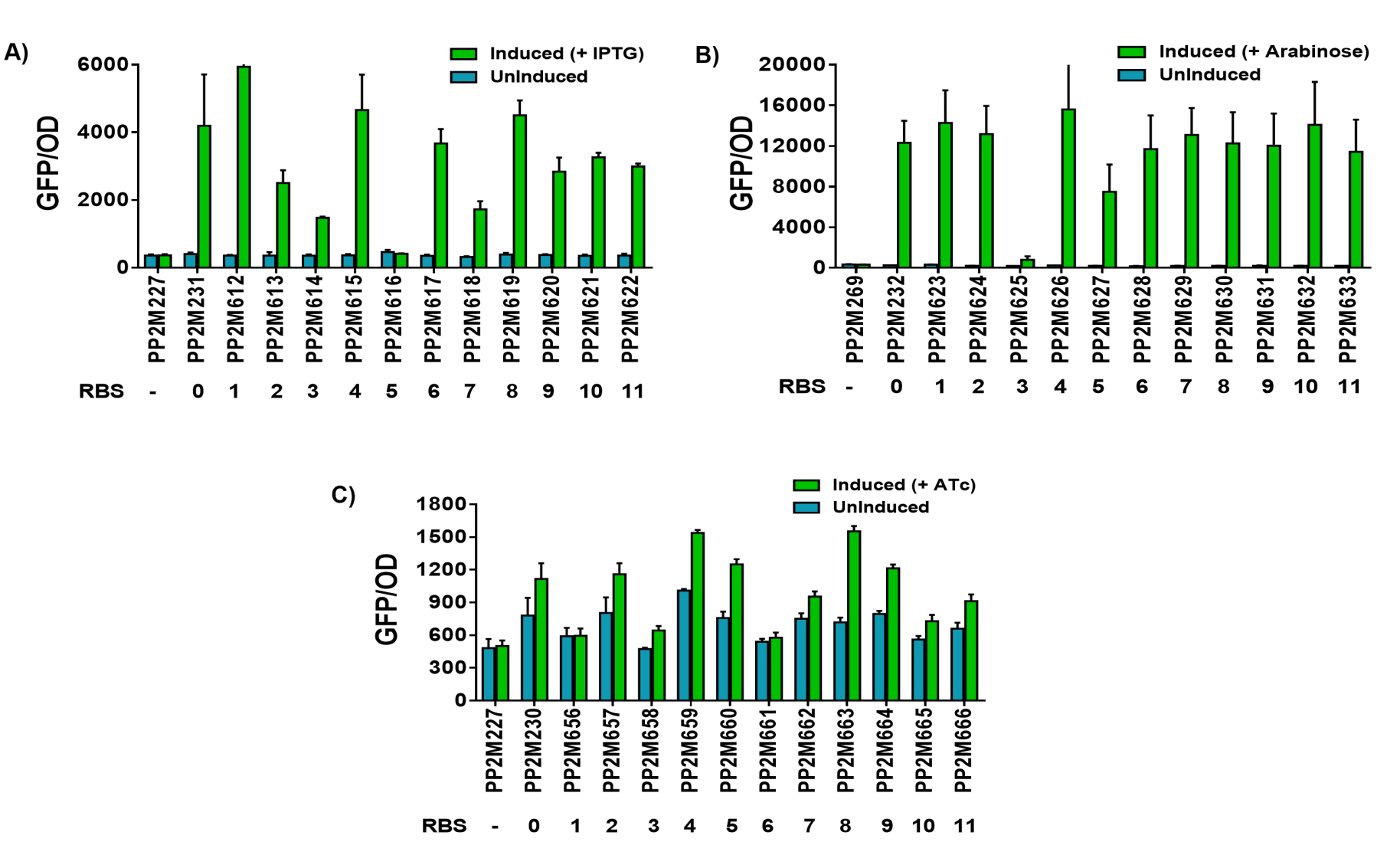
**

**Figure S1:** Comparative strengths of ribosome binding sites (RBSs). The selected RBS sequences (1 to 11) were taken from literature (Wang et al., 2018) and previously designed to modulate gene expression in P. putida based on Salis RBS calculator to cover a wide spectrum of expression levels. GFP activity assay was performed to compare the translational efficiency of twelve different RBSs in M2 **(A, B, C)** in M9 minimal media supplemented with xylose (0.5% w/v). All recombinant strains (harbouring RBS plasmids) were induced based on respective promoter systems, namely, P*_tac_* **(A)**, P*_bad_* **(B)**, and P*_tetR/tetA_* **(C)** at 0 hours. Levels of fluorescence for induced (green bar in graphs) and uninduced (no inducer was added, blue bar in graphs) cultures are shown. The strains harbouring empty vector were used as control. The number below each strain represents the corresponding RBS sequence under the control of a specific promoter system. The (-) sign is indicative of empty vector, therefore no RBS. For strains description and promoter-RBS combination refer **Table 1**. Data represent mean values of triplicate assays from at least three individual cultivations, and error bars represent standard deviations.

**
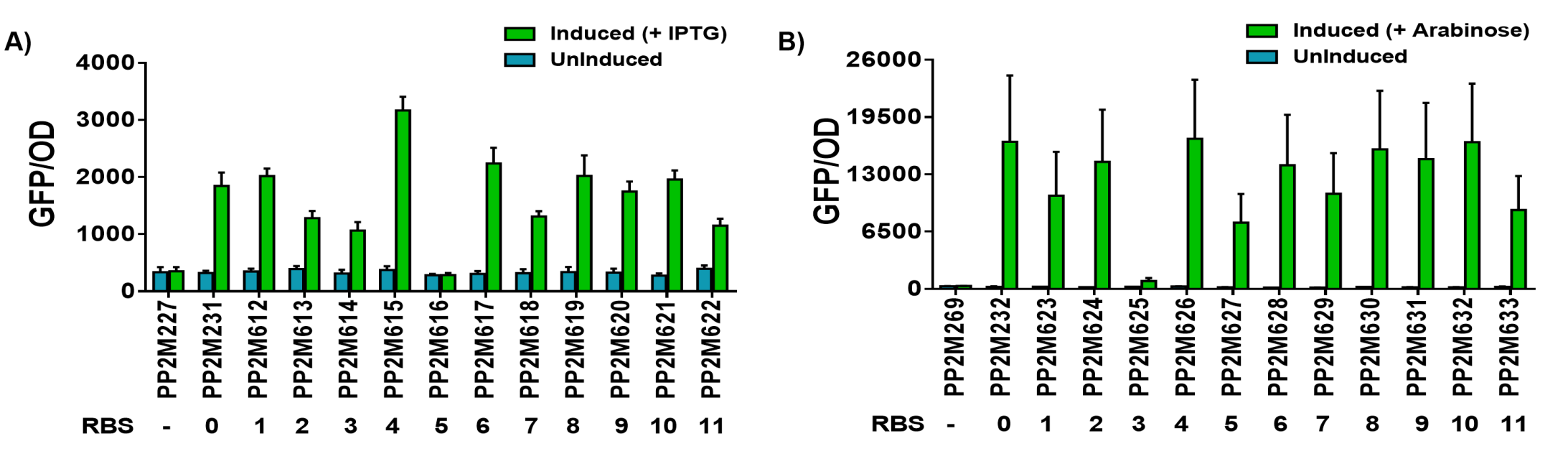
**

**Figure S2:** Comparative strengths of ribosome binding sites (RBSs). GFP activity assay was performed to compare the translational efficiency of twelve different RBSs in M2 **(A, B)** in M9 minimal media supplemented with arabinose (0.5% w/v). All recombinant strains (harbouring RBS plasmids) were induced based on respective promoter systems, namely, P*_tac_* **(A)**, and P*_bad_* **(B)**, at 0 hours. Levels of fluorescence for induced (green bar in graphs) and uninduced (no inducer was added, blue bar in graphs) cultures are shown. The strains harbouring empty vector were used as control. The number below each strain represents the corresponding RBS sequence under the control of a specific promoter system. The (-) sign is indicative of empty vector, therefore no RBS. For strains description and promoter-RBS combination refer **Table 1**. Data represent mean values of triplicate assays from at least three individual cultivations, and error bars represent standard deviations.

**
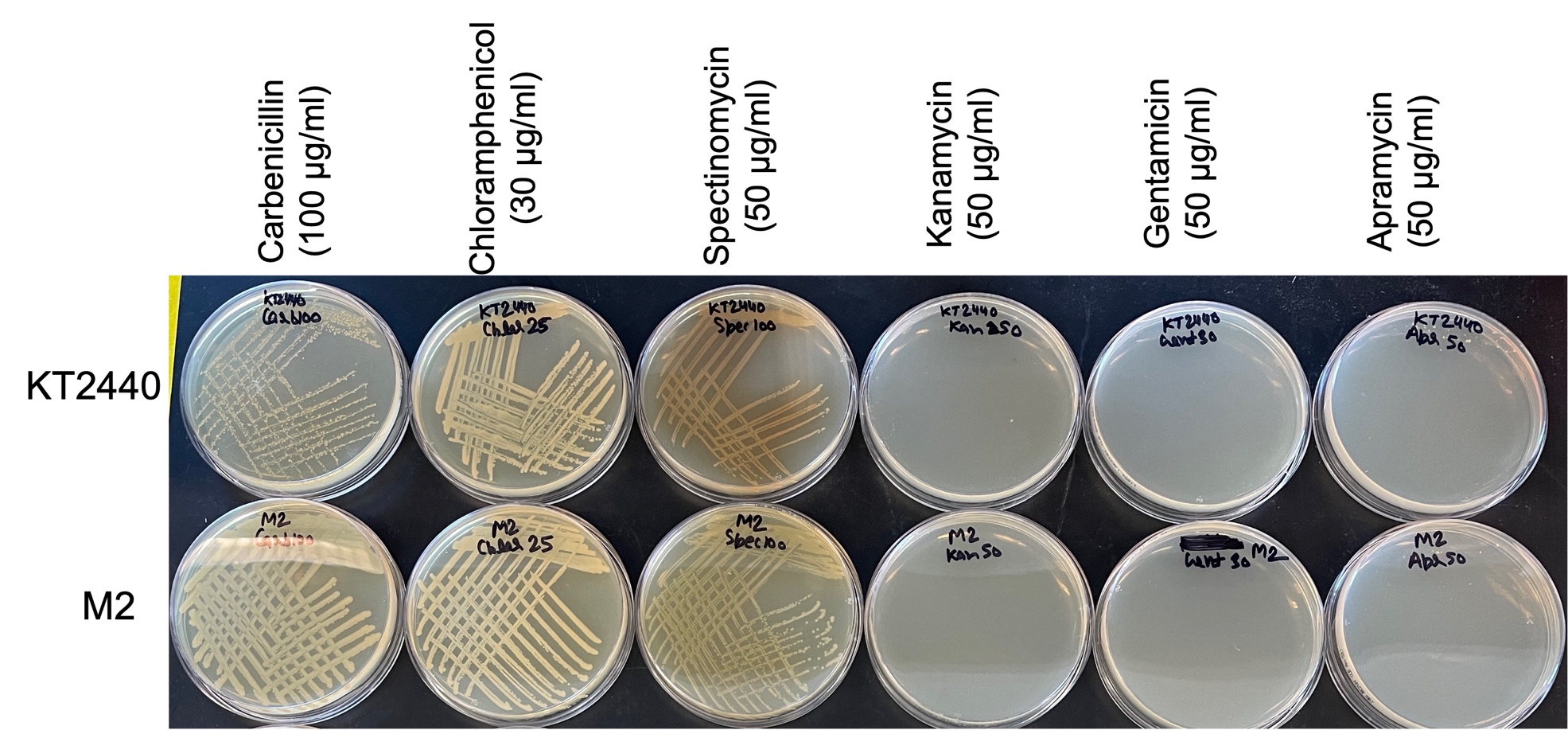
**

**Figure S3:** Testing antibiotic sensitivity of *P. putida* KT2440 and M2 against commonly used lab antibiotics. Both M2 and KT2440 were identified to be sensitive against Kanamycin, Gentamicin and Apramycin. Both KT2440 and M2 were identified to be resistant against Carbenicillin, Chloramphenicol and Spectinomycin.

**
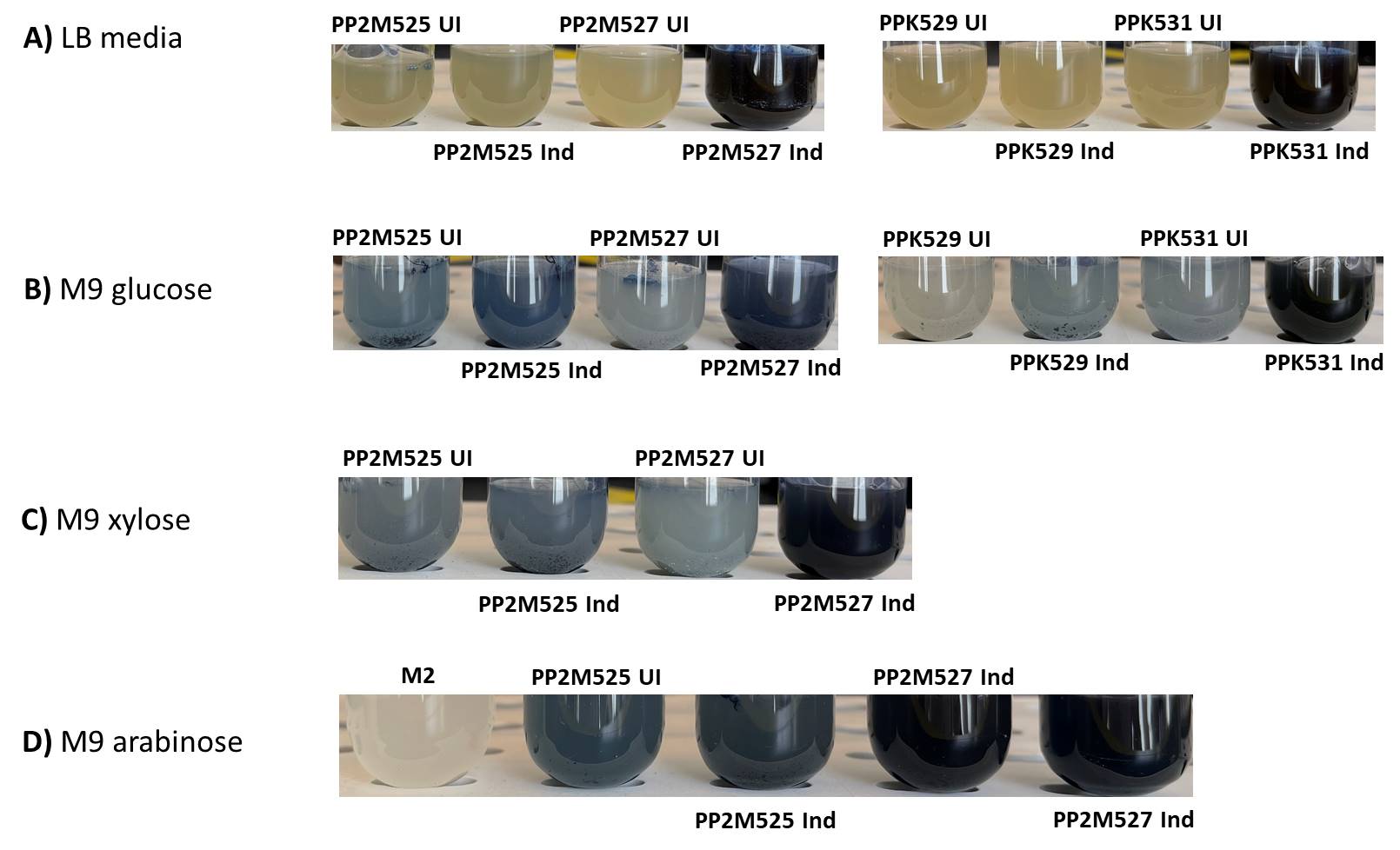
**

**Figure S4:** Indigoidine production in environmental isolate M2 in culture tubes after 24 hour of growth at 30 C (200 rpm) from complex media Luria bertani **(A)**, minimal medium M9 glucose **(B)**, M9 xylose **(C)** and M9 arabinose **(D)**. The recombinant strains expressed plasmid encoded heterologous genes *bpsA* from *S. lavendulae* and *sfp* from *B. subtilis* for conversion of glutamine to indigoidine under the control of either ATc-inducible or arabinose-inducible promoters. For strain description refer **Table 1**.


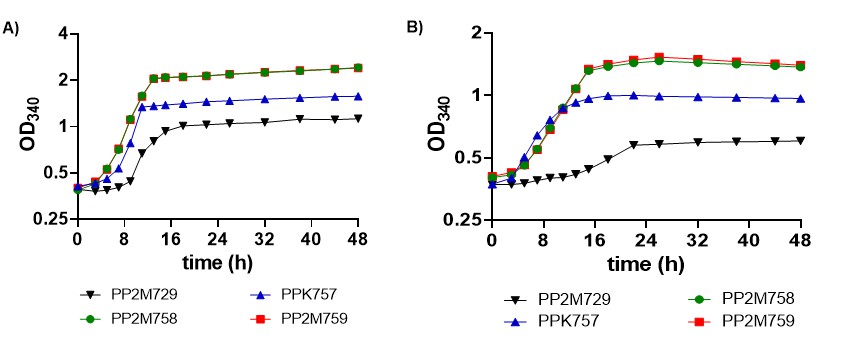


**Figure S5:** Kinetic assay curves for flaviolin producers at the wavelength of 340 nm in minimal medium with glucose (A) and xylose as C-source (B). Absorbance signal is directly proportional to the flaviolin production.

**
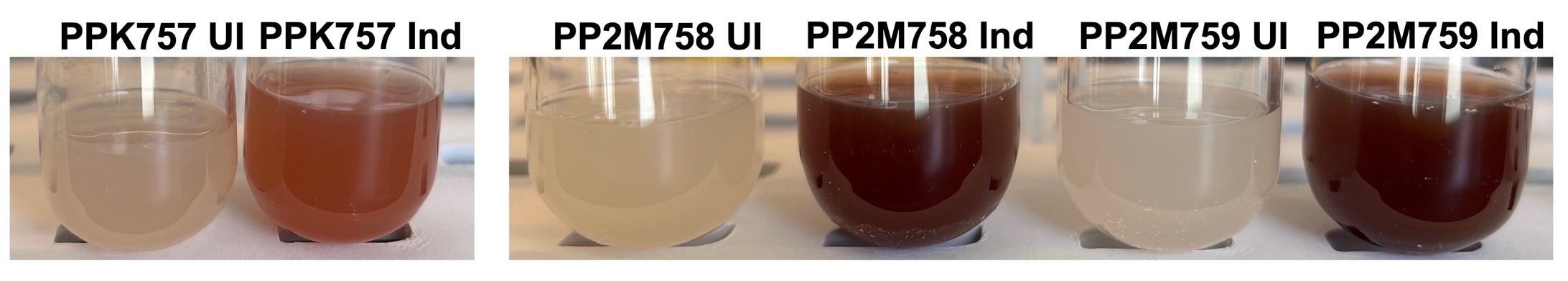
**

**Figure S6:** Flaviolin production in environmental isolate M2 (and KT2440) in culture tubes after 24 hour of growth at 30 C (200 rpm) with chromosomally integrated *rppA* using CRAGE technology in minimal medium glucose as C-source. For strain description refer **Table 1**.
